# Diagnosis of pine wilt disease using remote wireless sensing

**DOI:** 10.1371/journal.pone.0257900

**Published:** 2021-09-24

**Authors:** Sang-Kyu Jung, Seong Bean Park, Bong Sup Shim

**Affiliations:** 1 Bio. & Chemical Engineering, Hongik University, Sejong, S. Korea; 2 Research Laboratory, ECONNBIZ CO., LTD., Sejong, S. Korea; 3 Korea Forestry Promotion Institute, Seoul, S. Korea; RMIT University, AUSTRALIA

## Abstract

Pine wilt disease caused by *Bursaphelenchus xylophilus* is a major tree disease that threatens pine forests worldwide. To diagnose this disease, we developed battery-powered remote sensing devices capable of long-range (LoRa) communication and installed them in pine trees (*Pinus densiflora)* in Gyeongju and Ulsan, South Korea. Upon analyzing the collected tree sensing signals, which represented stem resistance, we found that the mean absolute deviation (MAD) of the sensing signals was useful for distinguishing between uninfected and infected trees. The MAD of infected trees was greater than that of uninfected trees from August of the year, and in the two-dimensional plane, consisting of the MAD value in July and that in October, the infected and uninfected trees were separated by the first-order boundary line generated using linear discriminant analysis. It was also observed that wood moisture content and precipitation affected MAD. This is the first study to diagnose pine wilt disease using remote sensors attached to trees.

## Introduction

Pine wilt disease (PWD) is one of the major plant diseases that, despite years of research and control efforts, constantly threaten pine forests in Japan, China, Canada, and Europe [1–4]. PWD was first reported in Japan in 1905, and has spread nationwide in Korea since it was first discovered in Busan in 1988 [1]. PWD is caused by the pine wood nematode, *Bursaphelenchus xylophilus*, which is transferred to trees by vector insects such as *Monochamus alternatus* and *Monochamus saltuarius* [1,5]. Once infection begins, the pine trees gradually dry from the top to the bottom and die [6].

Aerial photography and image analysis technologies are available to diagnose PWD. PWD or canopy decline due to PWD could be detected in color, false-colour/near-infrared (FCNI), hyper-spectral or (and) thermal images taken from a drone [7–12]. However, actual drone operations are limited owing to unsuitable weather conditions in forests, short drone flight times, and limited payload [13–15]. Real-time monitoring is currently unfeasible using aerial photographic analysis. Specifically, in forests containing both hardwoods and conifers, the diagnosis of PWD may be more difficult during fall and winter.

Wireless remote sensing technology is an alternative that can help predict the onset of PWD. There have been studies in which researchers measured stem water content using sensors attached to plants [16–18]. Changes in stem water content are an important symptom associated with PWD, and there is evidence that stem water content decreases as PWD progresses [5,19,20]. It is possible that changes in the water content inside pine trees depend on the cycle of night and day, and changes in the minerals and photosynthetic organic products that act as electrolytes may affect the internal stem resistance of the trees. Consequently, we anticipated that there would be a difference in the sensing signal patterns of healthy and infected trees.

In this study, we (1) developed a battery-powered remote sensing device, (2) attached the device to wild pine trees in a forest, and measured sensing data of the trees at regular intervals, (3) collected sensing data from a distance through long-range (LoRa) communication in real time, and (4) developed a technology to diagnose infected trees by performing statistical analysis of processed sensing signals. We have been collecting data since 2017 from sensing devices installed in multiple forest areas such as Gyeongju and Ulsan, where PWD occurs regularly and causes considerable damage to pine forests. For remote sensing, a LoRa network commercially built by SK Telecom (Seoul, South Korea) in 2017 was used to wirelessly collect sensing data from sensing devices in forest areas in real time. For reference, the lowest monthly rate in 2021 is 350 Korean won (US$ 0.31/month), which is very affordable.

Upon analyzing the collected sensing data, we found that there was a difference in the changes in the sensing signals of uninfected and infected pine trees, and that the mean absolute deviation (MAD) could be used to distinguish between the two classes. To the best of our knowledge, this is the first study in which PWD was diagnosed using remote sensors attached to trees. It is expected that this technology will be fully utilized for diagnosing plant diseases and monitoring the physiological growth characteristics of various plants.

## Materials and methods

### Test sites and trees

Sensing devices were installed in forests in Gyeongju and Ulsan, South Korea, which are areas that suffer severe damage due to PWD. The number of sensing devices installed in pine trees in Gyeongju and Ulsan was 75 and 65, respectively (1 sensing device per tree), and sensing data were collected from October 2018. Five trees in Gyeongju were excluded from the analysis due to device malfunction, device or tree loss, or device relocation. The sensing device ID, installation location, and inspection results are summarized in Table 1. The different locations of the installed sensors were measured using a Samsung Galaxy S9 equipped with a GPS module. Due to the mountainous nature of the area, there may have been some errors in the accuracy of the measured longitudes and latitudes.

**Table 1:** Sensing device installation location and tree inspection results in Gyeongju.

Tree number	Sensing device ID (LoRa modem unique ID)	Latitude	Longitude	PWD nematode inspection^a^	Wood chip moisture content (%)^b^	Visual observation (photographed)^c^
**1**	93702c1ffffe4f6b04	35.67781	129.41805	+ (found)	50.3 ± 3.5	Healthy
**2**	93702c1ffffe4f6b1c	35.67781	129.41802	-	N.A.	Healthy
**3**	93702c1ffffe4f6ac6	35.67787	129.41797	-	42.1 ± 1.7	Healthy
**4**	93702c1ffffe4f6ace	35.67792	129.41799	-	36.7 ± 2.3	Healthy
**5**	93702c1ffffe4f6bbe	35.67792	129.41799	+ (found)	32.1 ± 3.6	Dying
**6**	93702c1ffffe4f6c28	35.67793	129.41812	-	50.1 ± 1.1	Healthy
**7**	93702c1ffffe4f6c12	35.67775	129.41798	-	50.1 ± 1.4	Healthy
**8**	93702c1ffffe4f6b18	35.67779	129.41798	-	47.8 ± 2.5	Healthy
**9**	93702c1ffffe4f6c17	35.67787	129.41796	-	26.0 ± 5.5	Healthy
**10**	93702c1ffffe4f6c0d	35.67779	129.41798	+ (found)	29.2 ± 1.4	Dying
**11**	93702c1ffffe4f6b3f	35.67790	129.41795	-	50.7 ± 2.5	Healthy
**12**	93702c1ffffe4f6a94	35.67779	129.41796	+ (found)	29.6 ± 1.8	Dying
**13**	93702c1ffffe4f6c1d	35.67790	129.41828	-	47.7 ± 1.6	Healthy
**14**	93702c1ffffe4f6c55	35.67801	129.41790	-	49.8 ± 0.4	Healthy
**15**	93702c1ffffe4f6ad3	35.67779	129.41828	+ (found)	43.2 ± 2.6	Dying
**16**	93702c1ffffe4f6adc	35.67805	129.41797	-	31.6 ± 2.3	Healthy
**17**	93702c1ffffe4f6b2f	35.67801	129.41797	+ (found)	46.2 ± 1.7	Dying
**18**	93702c1ffffe4f6c14	35.67803	129.41797	-	46.4 ± 2.2	Healthy
**19**	93702c1ffffe4f6afc	35.67805	129.41791	-	46.9 ± 1.0	Healthy
**20**	93702c1ffffe4f6adb	35.67805	129.41781	+ (found)	34.2 ± 5.3	Dying
**21**	93702c1ffffe4f6af5	35.67802	129.41797	N.A.	42.9 ± 3.6	Healthy
**22**	93702c1ffffe4f6acc	35.67805	129.41781	-	35.2 ± 7.9	Healthy
**23**	93702c1ffffe4f6ad1	35.67807	129.41781	+ (found)	26.7 ± 1.2	Dying
**24**	93702c1ffffe4f6c53	35.67805	129.41779	-	26.6 ± 1.0	Dying
**26**	93702c1ffffe4f6a6e	35.67807	129.41780	+ (found)	24.7 ± 1.8	Healthy
**27**	93702c1ffffe4f6ab1	35.67807	129.41781	-	25.4 ± 1.1	Dying
**28**	93702c1ffffe4f6ac4	35.67807	129.41779	+ (found)	30.9 ± 3.1	Dying
**29**	93702c1ffffe4f6c1a	35.67810	129.41781	-	43.2 ± 0.7	Healthy
**30**	93702c1ffffe4f6c13	35.67805	129.41779	-	46.4 ± 1.3	Healthy
**31**	93702c1ffffe4f6b0b	35.67795	129.41788	-	44.6 ± 2.0	Healthy
**32**	93702c1ffffe4f6a8c	35.67808	129.41778	-	35.5 ± 6.9	Healthy
**33**	93702c1ffffe4f6adf	35.67812	129.41785	-	40.0 ± 4.7	Healthy
**34**	93702c1ffffe4f6a8b	35.67809	129.41783	-	47.8 ± 3.4	Healthy
**35**	93702c1ffffe4f6aef	35.67818	129.41777	-	41.9 ± 1.8	Healthy
**36**	93702c1ffffe4f6af6	35.67812	129.41783	+ (found)	40.4 ± 8.2	Dying
**37**	93702c1ffffe4f6b16	35.67823	129.41767	-	48.1 ± 7.0	Healthy
**38**	93702c1ffffe4f6c58	35.67799	129.41776	-	38.3 ± 2.2	Healthy
**39**	93702c1ffffe4f6a9d	35.67800	129.41777	-	39.0 ± 0.8	Healthy
**40**	93702c1ffffe4f6b89	35.67808	129.41774	-	45.1 ± 3.7	Healthy
**42**	93702c1ffffe4f6a6f	35.67806	129.41772	-	24.9 ± 2.4	Dying
**43**	93702c1ffffe4f6aa0	35.67810	129.41777	-	45.0 ± 1.4	Healthy
**45**	93702c1ffffe4f6b44	35.67823	129.41769	-	48.4 ± 3.0	Healthy
**47**	93702c1ffffe4f6b0a	35.67820	129.41770	-	41.9 ± 0.4	Healthy
**48**	93702c1ffffe4f6a9f	35.66887	129.42026	-	44.4 ± 1.6	Healthy
**49**	93702c1ffffe4f6af8	35.66887	129.42026	-	39.4 ± 2.9	Healthy
**50**	93702c1ffffe4f6c2c	35.66887	129.42026	+ (found)	39.4 ± 0.6	Healthy
**51**	93702c1ffffe4f6c2f	35.66887	129.42026	-	42.0 ± 3.7	Healthy
**53**	93702c1ffffe1cf906	35.66887	129.42026	-	43.0 ± 4.6	Healthy
**54**	93702c1ffffe4f6acf	35.66887	129.42026	-	40.2 ± 2.1	Healthy
**55**	93702c1ffffe4f6b11	35.66959	129.42285	-	39.3 ± 3.0	Healthy
**56**	93702c1ffffe1d18bc	35.66887	129.42026	-	37.3 ± 10.9	Healthy
**57**	93702c1ffffe4f6a80	35.66887	129.42026	-	37.4 ± 1.4	Healthy
**58**	93702c1ffffe4f6af0	35.66887	129.42026	-	47.1 ± 0.4	Healthy
**59**	93702c1ffffe4f6b42	35.66959	129.42285	-	45.9 ± 2.5	Healthy
**60**	93702c1ffffe4f6b0e	35.66867	129.43192	-	46.8 ± 0.5	Healthy
**61**	93702c1ffffe4f6c3c	35.66959	129.42285	-	40.9 ± 1.8	Healthy
**62**	93702c1ffffe4f6b23	35.66887	129.42026	-	48.2 ± 2.9	Healthy
**63**	93702c1ffffe4f6acd	35.66959	129.42285	-	45.5 ± 1.8	Healthy
**64**	93702c1ffffe4f6aeb	35.66887	129.42026	-	45.3 ± 3.9	Healthy
**65**	93702c1ffffe4f6aec	35.66887	129.42026	-	30.5 ± 2.6	Healthy
**66**	93702c1ffffe4f6c2a	35.66959	129.42285	-	48.0 ± 3.1	Healthy
**67**	93702c1ffffe4f6b09	35.66887	129.42026	+ (found)	45.4 ± 2.0	Dying
**68**	93702c1ffffe4f6c5b	35.66887	129.42026	-	45.0 ± 1.6	Healthy
**69**	93702c1ffffe4f6b1f	35.66887	129.42026	-	30.4 ± 2.1	Dying
**70**	93702c1ffffe4f6c56	35.66887	129.42026	-	52.2 ± 1.1	Healthy
**71**	93702c1ffffe4f6add	35.66887	129.42026	-	48.6 ± 1.7	Healthy
**72**	93702c1ffffe4f6b20	35.66887	129.42026	+ (found)	27.9 ± 2.5	Dying
**73**	93702c1ffffe4f6c22	35.66887	129.42026	+ (found)	38.0 ± 2.1	Dying
**74**	93702c1ffffe4f6ae3	35.66887	129.42026	-	46.2 ± 0.8	Healthy
**75**	93702c1ffffe4f6c1e	35.66887	129.42026	+ (found)	23.0 ± 0.9	Dying

^**a**^PWD nematode inspection was performed from July to September 2019.

^**b**^Wood chips were collected in September 2019.

^**c**^Visual observation was carried out in January 2020.

### Pine wilt disease infection examination

To inspect the infection of the pine wood nematode (*B*. *xylophilus*) in trees, wood chips were collected using a drill from July to September 2019 in Gyeongju, and in January 2020 in Ulsan. The wood chips were soaked in water to settle, and the precipitate was observed with a polarizing microscope (DE/DM500, Leica Microsystems) to confirm the presence of *B*. *xylophilus*. Thus, it was confirmed that 15 out of 75 trees were infected in Gyeongju and 3 out of 65 trees were infected in Ulsan. All test trees were manually inspected and photographed to record any discoloration and/or defoliation (S1 File). If significant discoloration or defoliation was observed throughout the tree, it was identified as a dying tree. Every tree infected with *B*. *xylophilus* was properly cut and removed by responsible government agents until February 2020 to prevent the spread of PWD infection.

### Wood chip moisture content analysis

Wood chips were collected using an electric drill. The moisture content was measured using a moisture analyzer (MB90, Ohaus, USA). One gram of wood chips was placed on an aluminum plate and heated until the weight stopped changing. The difference between the initial and final weights was measured to calculate moisture content. Measurements were performed three times, and the moisture content was presented as the mean and standard deviation.

### Data processing and statistical analysis

A program was developed using VB.NET for data processing and analysis. The processing order was as follows. Firstly, the sensor data stored in MySQL were downloaded; secondly, redundant data and some noise data were removed, and sorting was performed over time. Thirdly, baseline values were calculated by applying a median filter. Fourthly, deviation and the mean absolute deviation (MAD; the average of the absolute deviations) were calculated. Fifthly, charts were created on a monthly and annual basis and used for analysis and data inspection. All the data and charts generated through processing were inspected by humans to ensure that there were no abnormalities. For statistical analysis, ANOVA on ranks followed by Dunn’s post-hoc test was performed using SigmaPlot 12, and linear discriminant analysis was performed using MATLAB (S2 File). In bar graphs, errors were expressed as 95% confidence intervals.

## Results and discussion

### Development of a sensing device

**Fig 1:**
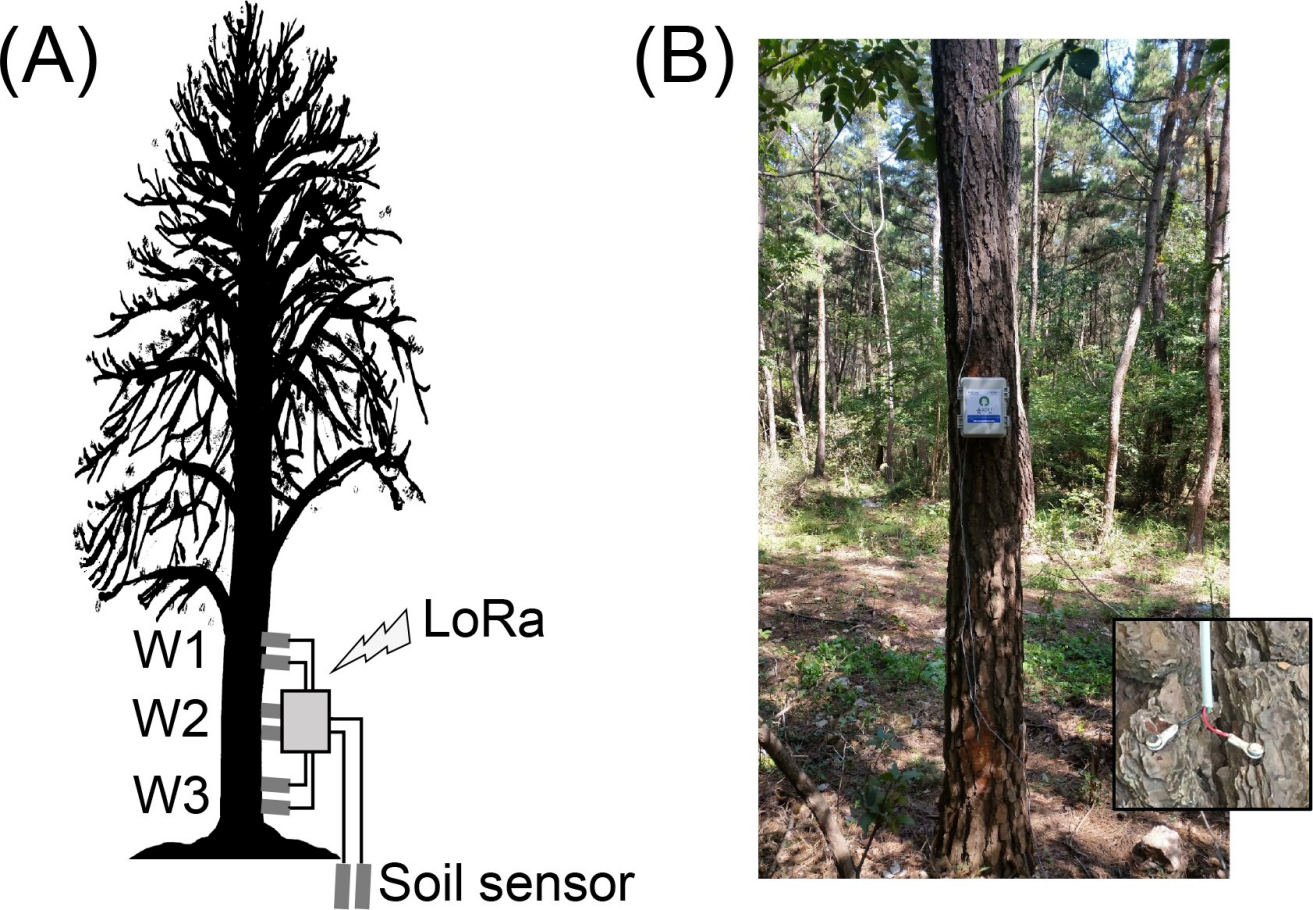
Installation of a battery powered remote sensing device. (A) Schematic diagram of the installation of sensor electrodes (W1, W2, and W3) and soil sensor. The measured data were transmitted via long-range (LoRa) communication. (B) Actual scene where the sensing device was installed and photo of electrode installation.

The sensing device consisted of a microcontroller (Arduino Pro Mini 328P), an alkaline battery pack, a LoRa modem, and a sensor module. Three pairs of stainless-steel nails were inserted at different locations on trees, and the distance between each pair of nails was 3 cm (Fig 1). By connecting the electrodes to the voltage divider circuit of the sensing device, the stem resistance between the electrodes was converted to a voltage and read through a 10-bit analog input pin of the microcontroller. The height of the W1 sensor installed at the highest point was approximately 2.5 m from the ground; sensors W2 and W3 were installed at lower positions. To measure the moisture content of the soil, custom electrodes were installed in the soil. A temperature and humidity sensor (DHT-11) were also included in the sensing device to monitor the atmospheric environment.

The sensing device was programmed to enter sleep mode, except for a short period during sensing and LoRa communication in order to operate on a battery for a long time. Sensing and data transmission were performed at 1 h intervals. The data sent from the LoRa modem was propagated to the SK Telecom (Seoul, South Korea) LoRa network, processed by the base station, and finally collected in our MySQL server.

The resistance between electrodes can be measured as a voltage using a simple voltage divider circuit. The tree sensor value measured with a 10-bit analog to digital converter (ADC) ranged from 0 to 1023. There was a correlation between the resistance between the electrodes (R kΩ) and the 10-bit analog sensor value:

$$\mathrm{Sensor}\;\mathrm{value}=1023\times \frac{R}{R_{1}+R},\;\mathrm{where}\;R_{1}=10\;k\Omega$$ (1)

This equation indicates that the resistance decreases with a decrease in the sensor value. Conversely, if the resistance is infinite or has a large value, the sensor value reaches a maximum of 1023. We tested multiple carbon composition resistors having various resistance values with our sensing module and confirmed that the calculated resistance value was the same as the actual resistance value (R^2^ = 1.000).

### Remote sensing of trees and environmental monitoring

**Fig 2:**
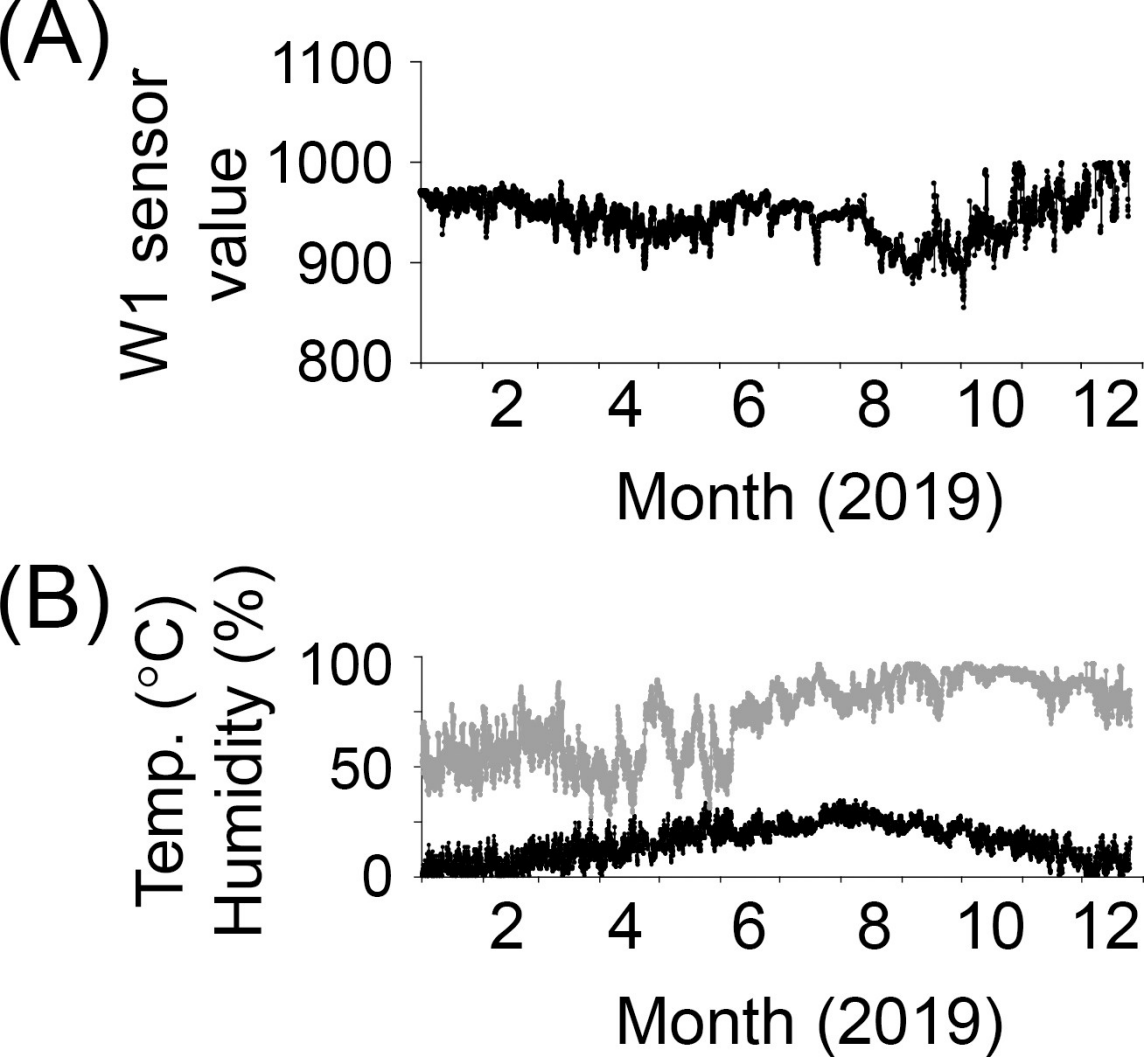
Actual measurement of a remote sensing device in Gyeongju. (A) Changes in W1 sensor value measured in one tree for a year. (B) Changes in temperature (black line) and humidity (gray line) where the sensing device was installed.

**Fig 3:**
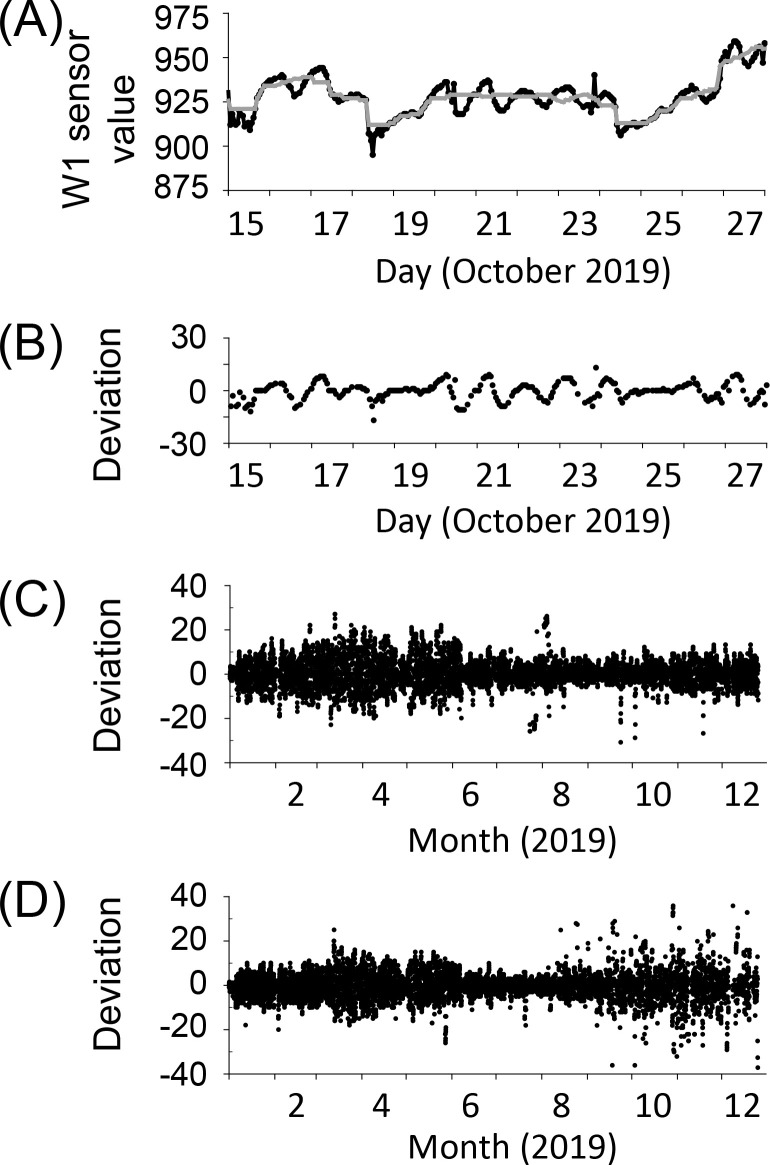
Sensor signal processing and deviation computed in Gyeongju. (A) Generation of baseline sensor values (gray line) from raw sensor values (black line) by applying a median filter. (B) Computed deviation, i.e., difference between the baseline and measured sensor values. (C) Annual change in the deviation of an uninfected tree. (D) Annual change in the deviation of an infected tree.

The annual change in sensing data collected from one infected tree in Gyeongju in 2019 and the short-term changes over approximately two weeks are shown in Figs 2A and 3A, respectively. Fig 2B shows the temperature and humidity changes at the location where the sensing device was installed.

In Gyeongju, the sensing value of W1 was generally lowest in August and September and tended to be high in winter (Fig 2A). The tree sensing signal vibrated in a cycle 24 h a day, with the highest value (i.e., the highest resistance) between 4 am and 6 am; the signal then decreased, with the sensor value at the lowest (i.e., the lowest resistance) in the afternoon between 1 pm and 3 pm (Fig 3A). In a study that measured the sap flow rate of a maple tree, there was almost no sap flow from midnight to 6 am, and the flow rate had a maximum value of 100 to 150 mm/h at approximately 2 pm [17]. Similarly, in an experiment with a tomato tree, there was almost no sap flow from midnight to 6 am, and the sap flow rate had a maximum value of approximately 1,400 mm/h from 12 pm to 4 pm [16]. The atmospheric temperature might have affected our sensing measurements to some extent; however, as the sap flow rate increases, the stem water content is expected to increase, and consequently, the resistance is expected to decrease. Therefore, our experimental results can be considered to be in good agreement with those in other reports.

### Classification of infected and uninfected trees by mean absolute deviation (MAD) of sensing signals

To diagnose PWD based on these sensing signals, the deviation and MAD were calculated by processing the signals. Firstly, baseline values (shown in gray) were calculated by applying a median filter to remove the vibrating pattern (Fig 3A). Because the sensing signal changed in a daily cycle and sensing was set to be performed once per hour, a total of 25 sensing data (one center point + 12 data received before the center point + 12 data received after the center point) corresponding to approximately 24 h of sensing data were used to compute the median value. However, various factors (such as the mountain region, weather, and installation location) appeared to lower the reception rate, and 3–6 of the expected 24 data were not received. Therefore, the actual data used for computing the median value was slightly larger than the 24-h data. Then, the MD, which is the difference between the sensor and baseline values, was calculated (Fig 3B). An examples of annual deviation changes of an infected and uninfected tree in Gyeongju are shown in Fig 3C and 3D, respectively.

**Fig 4:**
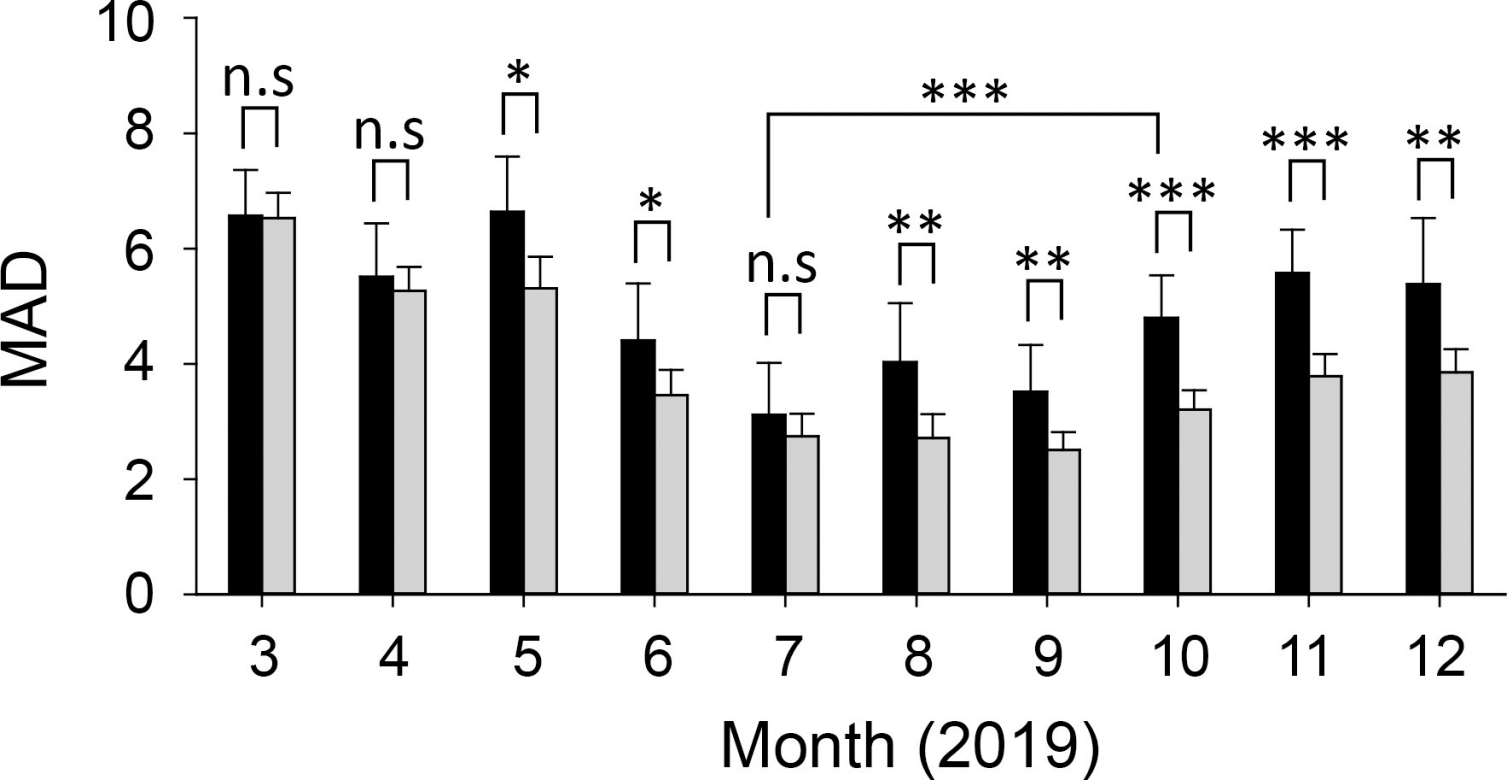
Monthly mean absolute deviation (MAD) of uninfected and infected trees in Gyeongju. * *p* < 0.05; ** *p* < 0.01; *** *p* < 0.001; *p*-value, one-way ANOVA on ranks. n.s: Not significant. The bars and error bars stand for means and 95% confidence intervals, respectively.

We found that both infected and uninfected trees exhibited a lower MAD value in the summer (July to September) than in other seasons (Fig 4). Especially in July, the MAD was at the lowest level during the year; moreover, the MADs of the infected and uninfected trees were similar. However, there were differences in the MAD changes of the uninfected and infected trees, especially after August; the MAD of infected trees was higher than that of uninfected trees (ANOVA on ranks, *p*-value < 0.01). The MAD value gradually increased from September, but the increase was larger in infected trees than in uninfected trees.

**Fig 5:**
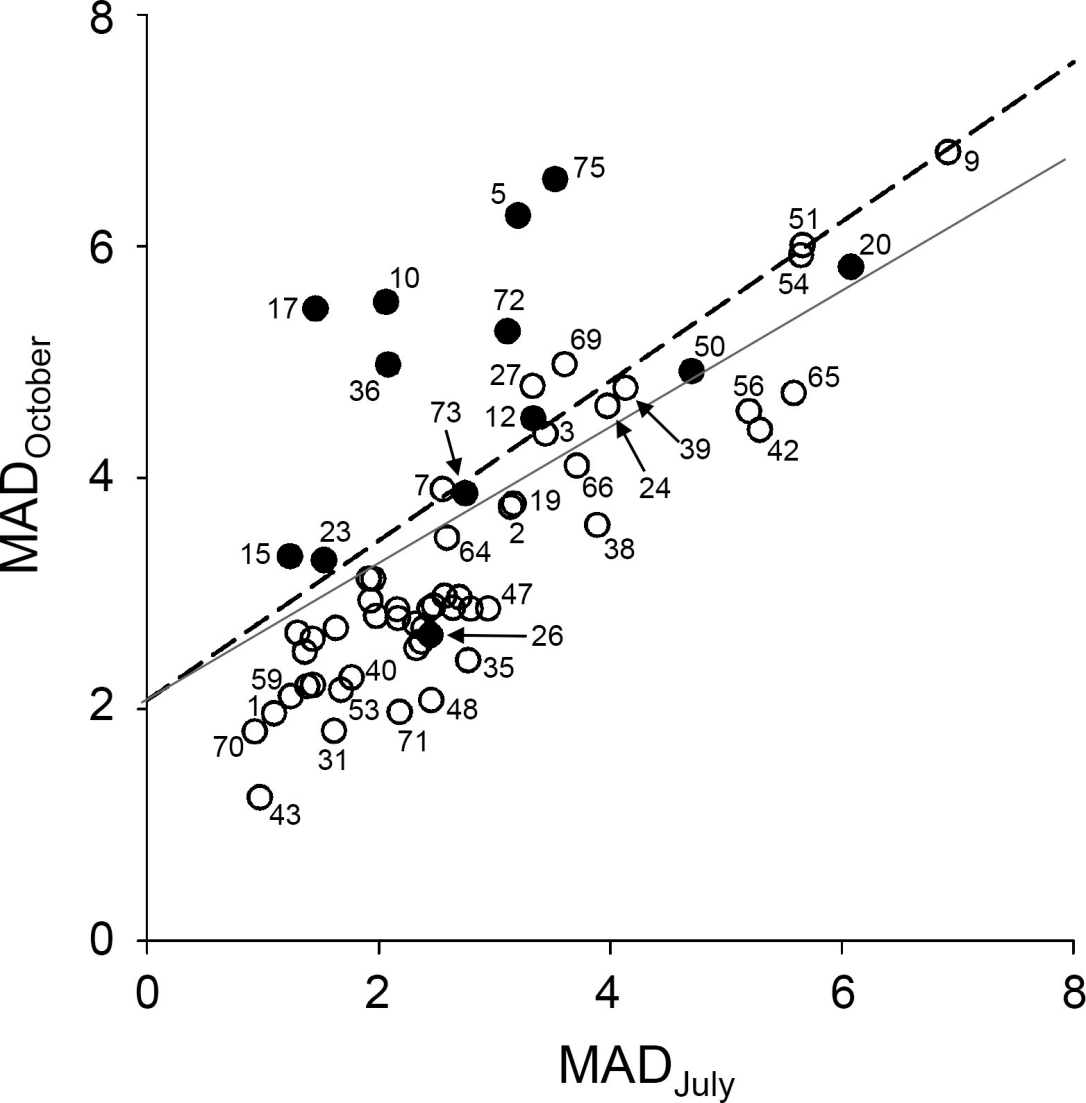
Classification of infected and uninfected trees by MAD values. Sample size: *n* = 13 for infected trees (closed dots); *n* = 50 for uninfected trees (open dots) for July and October in Gyeongju. Black dashed line: Separation line generated using linear discriminant analysis; gray line: Custom separation line.

A two-dimensional plane was constructed with MAD in July (MAD_July_) and MAD in October (MAD_October_) as the x and y axes, respectively, and the positions of individual trees were marked with dots (Fig 5). Surprisingly, the infected and uninfected trees displayed in the two-dimensional plan could be divided into two regions. The linear equation obtained by performing linear discriminant analysis was MAD_October_ = 2.0791 + 0.6888 × MAD_July_. When infection was diagnosed based on this separation line, the true positive accuracy was 70% (9 out of 13), and true negative accuracy was 94% (47 out of 50). However, when the optimized custom separation line was set to maximize the true positive accuracy, the true positive accuracy was 92.9% (12 of 13), and the true negative accuracy was 82.0% (42 of 50).

**Fig 6:**
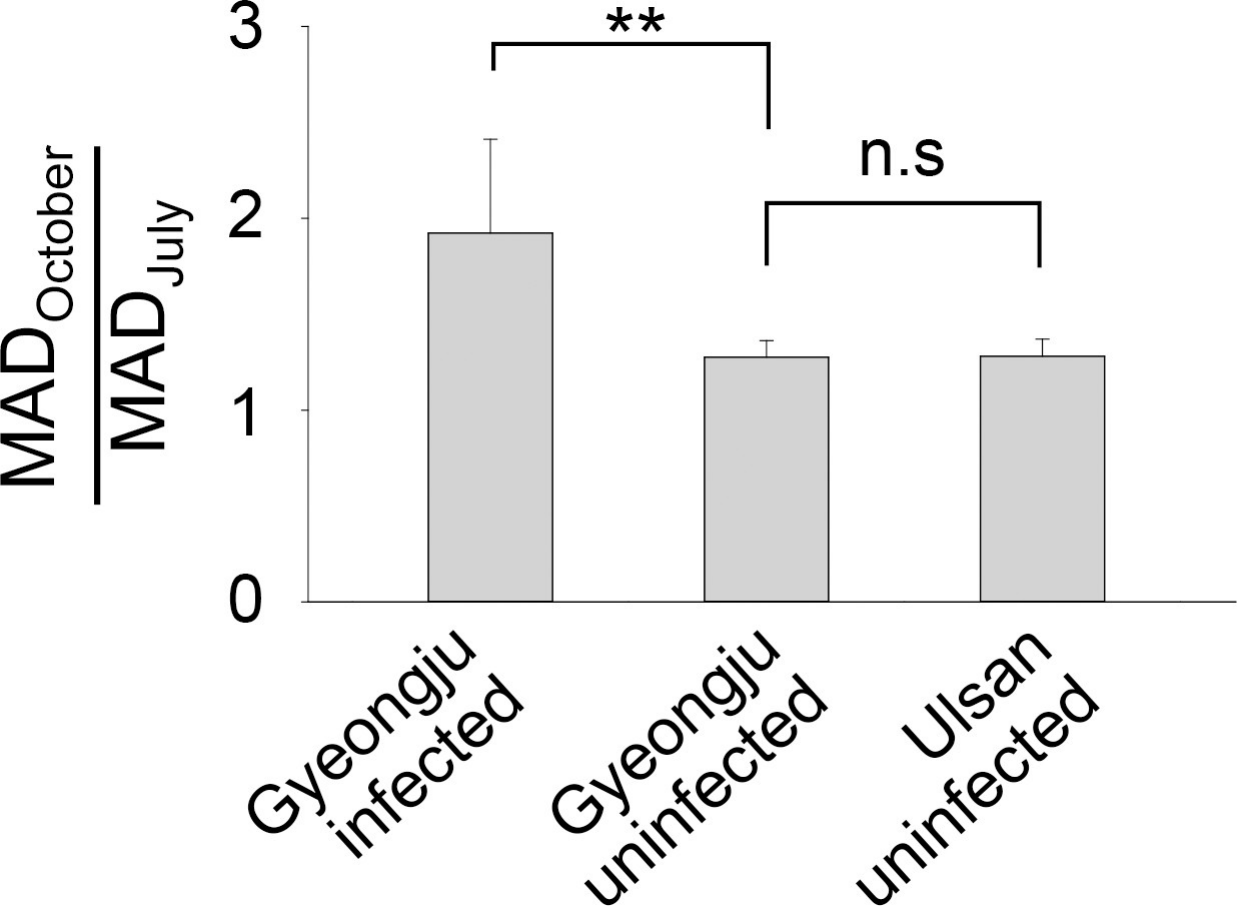
Difference in the ratio of MAD_October_ and MAD_July_ between infected and uninfected trees. Sample size: *n* = 13 for Gyeongju infected; *n* = 50 for Gyeongju uninfected; *n* = 45 for Ulsan uninfected. ** *p* < 0.01; *p*-value, one-way ANOVA on ranks. n.s: Not significant. The bars and error bars stand for means and 95% confidence intervals, respectively.

Since infected and uninfected trees were located above and below the separation line, respectively, in the two-dimensional plane, the average MAD_October_/MAD_July_ value (MAD ratio) of the infected trees was larger than that of the uninfected trees. The calculated MAD ratio of the infected trees was 1.924 ± 0.489 (mean ± 95% confidence interval), whereas that of the uninfected trees was 1.276 ± 0.087 (ANOVA on ranks, *p*-value = 0.005) (Fig 6). In addition, uninfected trees in Gyeongju and Ulsan had similar MAD ratios.

### Correlation between wood chip moisture content and MAD

**Fig 7:**
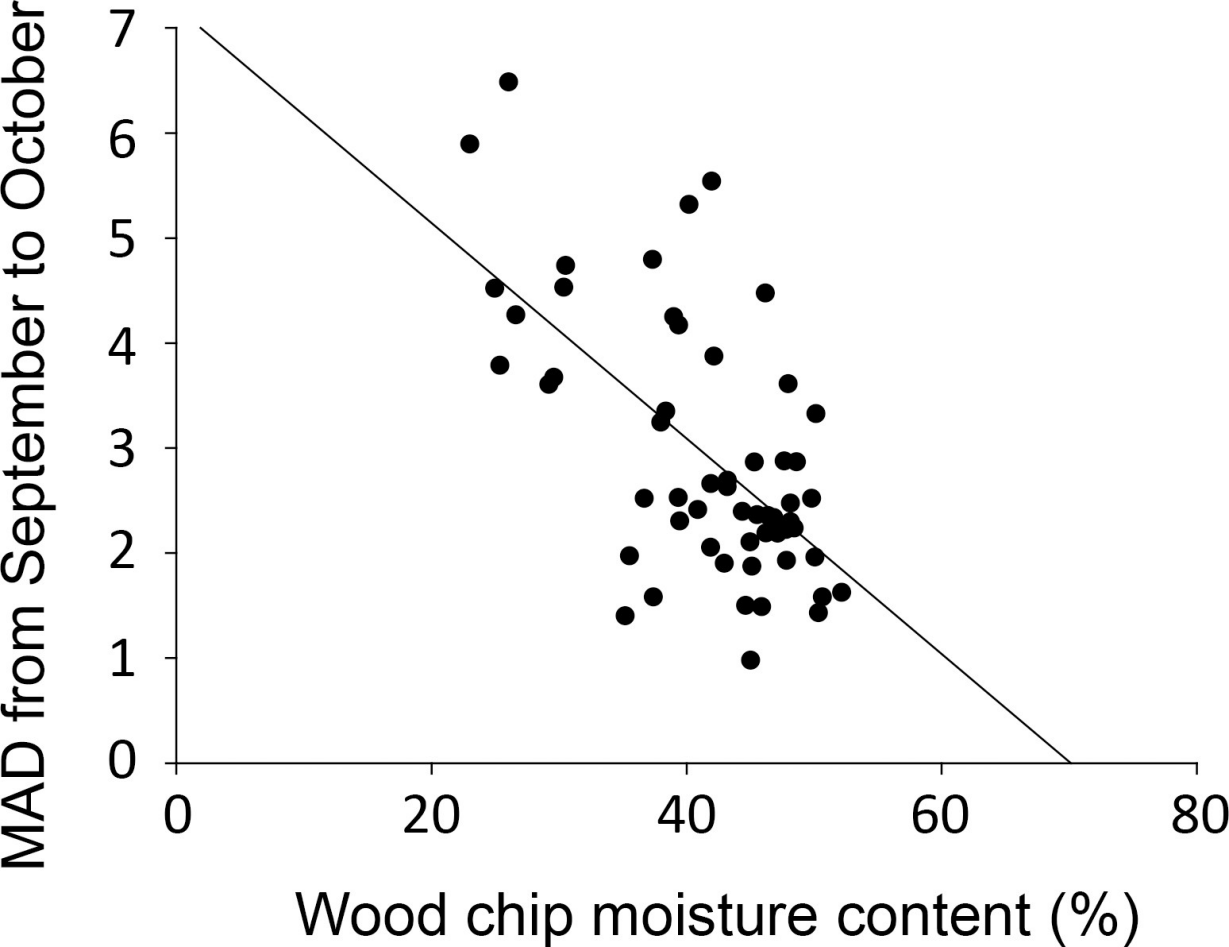
Correlation between wood chip moisture content and MAD from September and October. Wood chips were collected in September 2019. The bars and error bars stand for means and 95% confidence intervals, respectively.

To determine the cause of the change in the MAD value, we collected wood chips and analyzed their moisture content (Table 1). We observed that there was a linear relationship between the MAD and the moisture content of wood chips collected for September (regression coefficient R = 0.5511; S1 Fig). The linearity improved when data for the month of October was added (regression coefficient R = 0.6176) (Fig 7). It was observed that MAD decreased with increasing moisture content. PWD-infected pine trees generally tend to have a lower moisture content than healthy trees [5,19,20]. In our analysis, the wood chip moisture content of the infected trees was 16.6% lower than that of the uninfected trees, and the difference was statistically significant (ANOVA on ranks, *p* = 0.004; S2 Fig). Therefore, these results imply that the change in moisture content in infected trees had some effect on MAD.

### Further discussion on the diagnosis of pine wilt disease

The separation of two groups of uninfected and infected trees with a simple separation line sometimes led to misclassification, especially for trees located near the boundary region (Fig 5). Since the internal stem structure of each tree is different, the sensing value may vary depending on the location of the sensor electrode. Moreover, the time of infection and disease progression are different for individual trees, as are the physiological characteristics. They might have had an effect on the accuracy of the classification. To increase the accuracy, new methods should be developed to reduce the variance of sensor values and MADs between similar different trees. In particular, the installation of sensor electrodes is important, and it may be helpful to develop a method for detecting a sensor value within an appropriate range by installing the sensor electrodes at an optimal installation position.

Trees 26 and 50 in Gyeongju are in a group of uninfected trees below the separation line despite being infected. However, they were identified as healthy trees through visual observation (S1 File). In addition, Trees 24, 27, and 69 were found to be dying trees, although pine wood nematode (*B*. *xylophilus*) was not found in the wood chip examination. Perhaps, pine wood nematode could have been found if additional chips were collected and analyzed from these trees. At least, it is still possible to distinguish between healthy and dying trees through the separation line in the two-dimensional plane represented by MAD_July_ and MAD_October_. By calculating the average MAD_October_ and MAD_July_ values of multiple trees, it is possible to accurately detect whether PWD infection had occurred, or trees were dying in the area where the sensing devices were installed.

**Fig 8:**
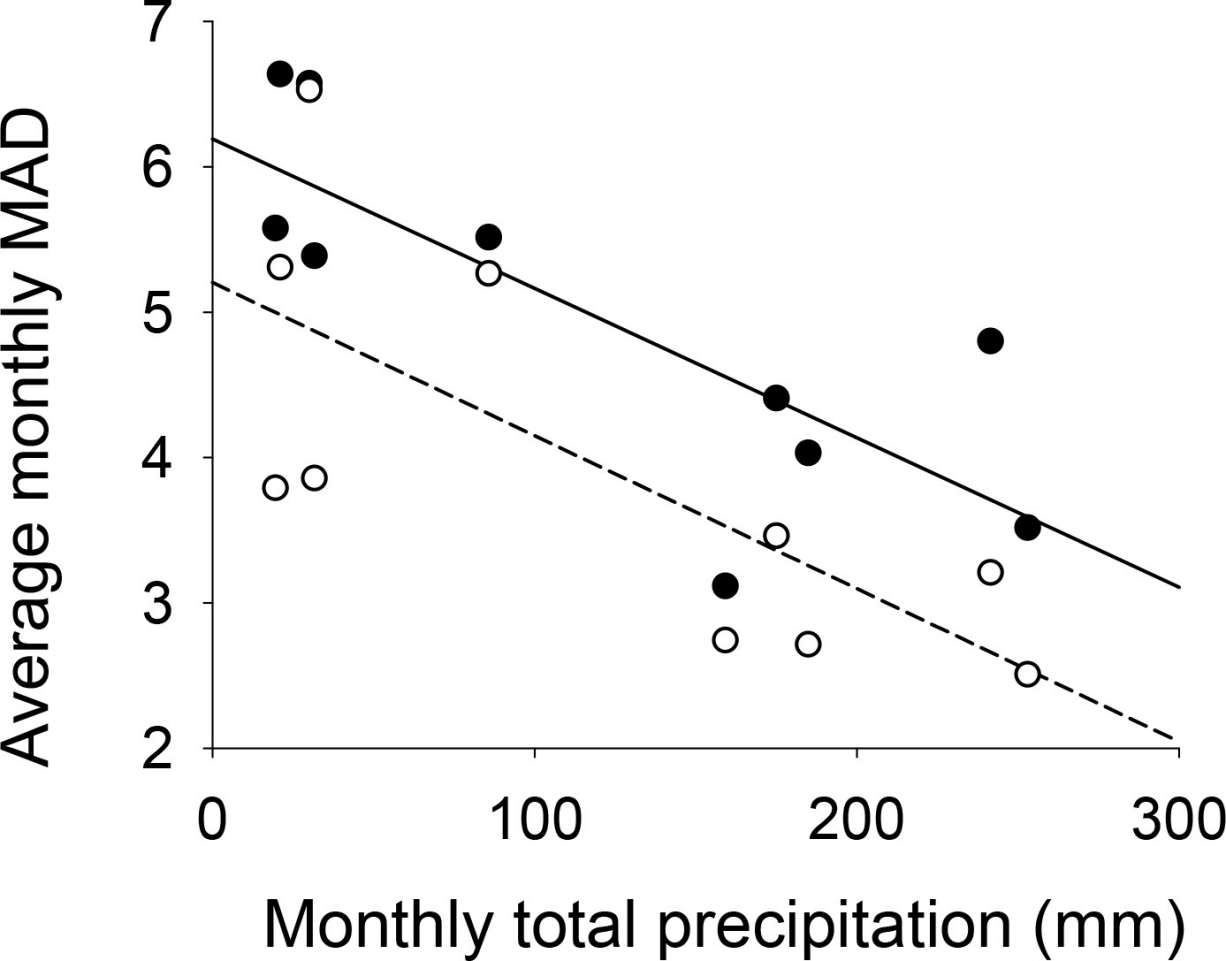
Correlation between average monthly MAD and monthly total precipitation from March to December. Closed points: Infected trees; Open points: Uninfected trees; Solid line: Regression line for infected tress; Dashed line: Regression line for uninfected trees. Each dot represents the total precipitation and the average MAD of trees for a specific month.

For both uninfected and infected trees, the MAD value was lowest in the summer, and the difference in MAD values of uninfected and infected trees was small; however, this difference increased from October, after the summer. Further research is needed, but our analysis results suggest that even the infected trees remained somewhat healthy in the summer, owing to sufficient light intensity and rainfall; it is sometimes difficult to observe any visible signs of infection (eg brown leaves) in the summer. In fact, weather data related to precipitation (S3 Fig) were compared to the average MAD of trees (Fig 4). Notably, as shown in Fig 8, there was a fairly strong negative correlation between the monthly total precipitation and the average monthly MAD of trees (regression coefficient R = 0.7988 for infected trees; R = 0.7337 for uninfected trees). For both infected and uninfected trees, the smaller the monthly total precipitation, the larger was the average monthly MAD value. These results implied that infected trees were more vulnerable than uninfected trees during the periods of low precipitation.

In this study, we discovered MAD as a key feature of time-series sensing data for PWD infection diagnosis. We also investigated other features such as the mean sensor value, but they were not as successful as MAD. Similar to MAD, the ratio between the mean sensor values in July and October of individual trees was also calculated. The ratio was then used to investigate whether there was a difference between the infected and uninfected trees. However, as shown in S4 Fig, there was no statistically significant difference between the values (ANOVA on ranks, *p* = 0.134). Nevertheless, it is still necessary to compute and study various features from the time-series data collected. Since the number of infected trees is not large, it may be more promising to find additional key features that distinguish infected and uninfected trees through dimension reduction techniques such as principal component analysis (PCA), linear discriminant analysis (LDA), and t-distributed stochastic neighbor embedding (T-SNE) than by artificial intelligence learning. The current method for diagnosing using the July and October MAD values is time-consuming in terms of an early diagnosis of PWD. New key features in the future are expected to help a faster diagnosis of PWD.

We not only fabricated a low-power remote sensing device capable of LoRa communication but also developed a novel technology to diagnose PWD by processing the sensor signals and calculating the MAD values. It is notable that existing disease diagnosis technology using sensors for humans and animals has been expanded to the plant domain. We expect this technology to be applied to the monitoring of physiological characteristics of various plants and the diagnosis of diseases, including fire blight disease, which causes great damage to orchard farms.

## Supporting information

S1 Fig Correlation between wood chip moisture content and MAD for September. (TIF)

S2 Fig Comparison of wood chip moisture content between infected and infected trees. (TIF)

S3 Fig Past weather data on the number of days of precipitation and total precipitation in Gyeongju. Data from the Open Weather Data Service of the Korea Meteorological Administration (data.kma.go.kr). The number of days of precipitation is defined as the number of days with a daily precipitation of 0.1 mm or more.
(TIF)

S4 Fig Difference in the ratio of mean sensor values for October and July for infected and uninfected trees in Gyeongju. Sample size: *n* = 14 for infected; *n* = 51 for uninfected. n.s: not significant (one-way ANOVA on ranks). The bars and error bars stand for means and 95% confidence intervals, respectively.
(TIF)

S1 File Photographs of the test trees in Gyeongju for visual observation. Photos were taken in January 2020.
(PDF)

S2 File MATLAB source code for discriminant analysis. (PDF)
