# Supplementary figures and images for: Diagnosis of pine wilt disease using remote wireless sensing
Source: PLoS One. 2021 Sep 24;16(9):e0257900. doi: 10.1371/journal.pone.0257900 (PMC8462718; doi:10.1371/journal.pone.0257900)

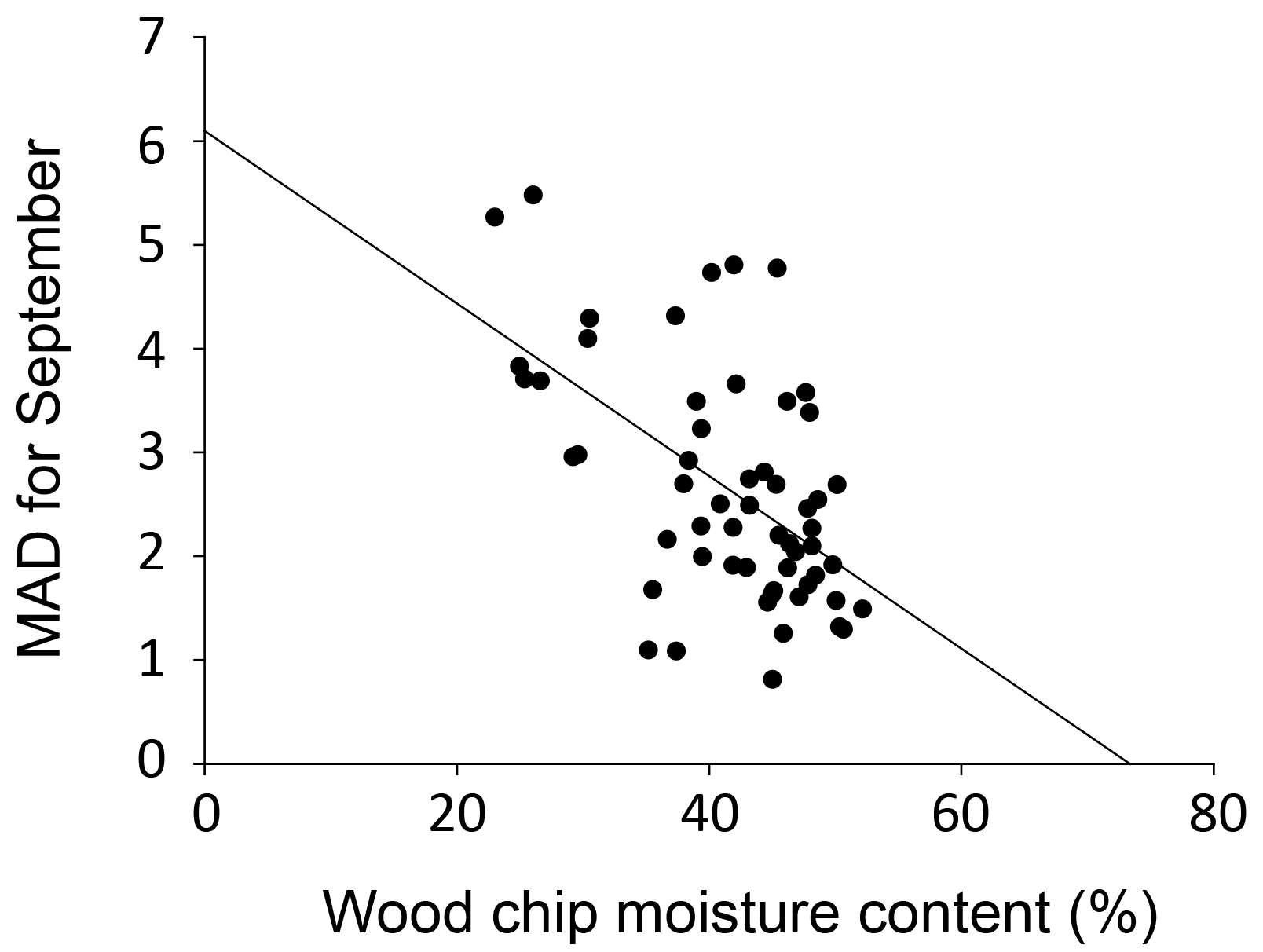

Supplement: S1 Fig — (TIF) [file pone.0257900.s001.tif]

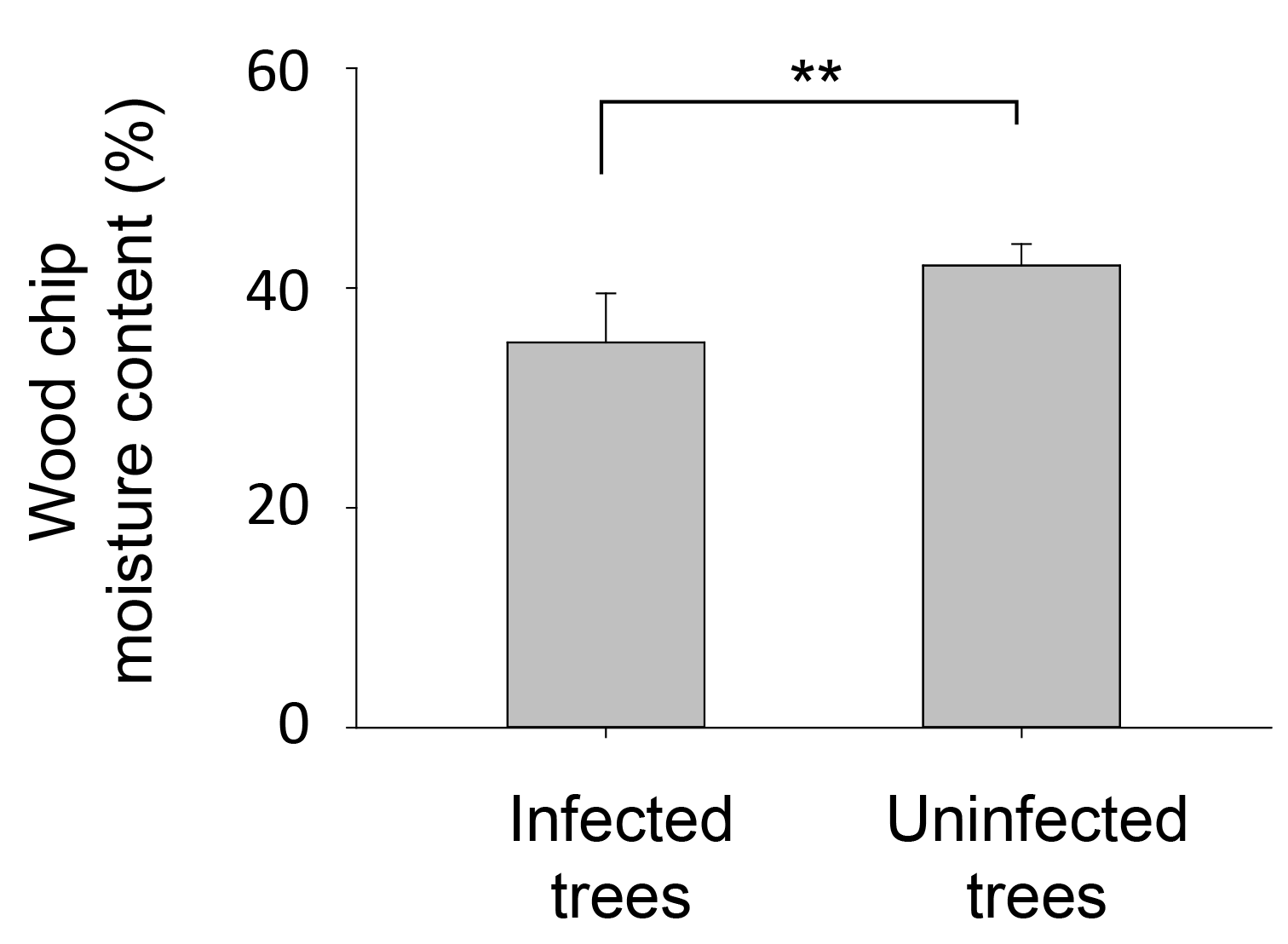

Supplement: S2 Fig — (TIF) [file pone.0257900.s002.tif]

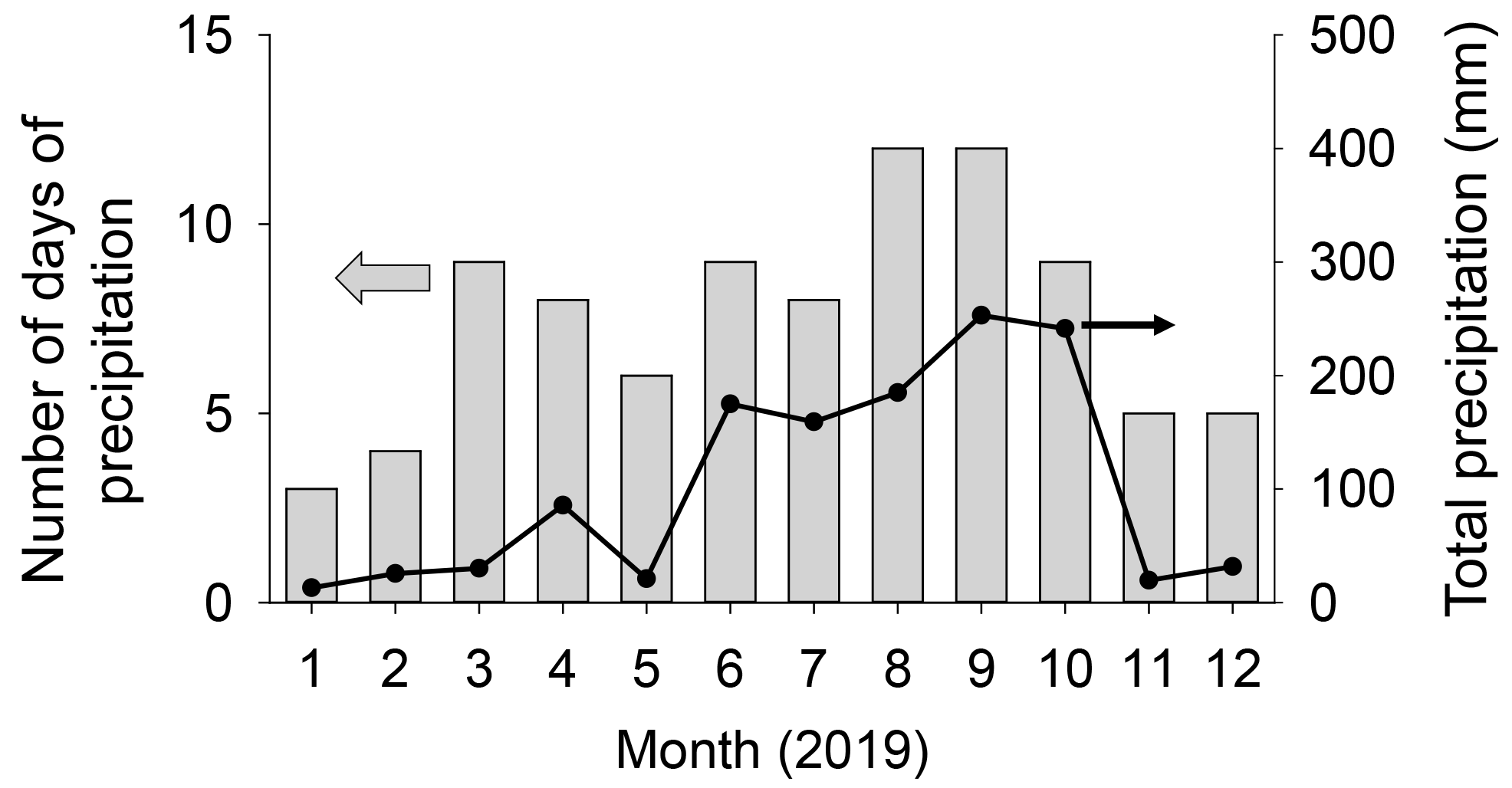

Supplement: S3 Fig — Data from the Open Weather Data Service of the Korea Meteorological Administration (data.kma.go.kr). The number of days of precipitation is defined as the number of days with a daily precipitation of 0.1 mm or more. (TIF) [file pone.0257900.s003.tif]

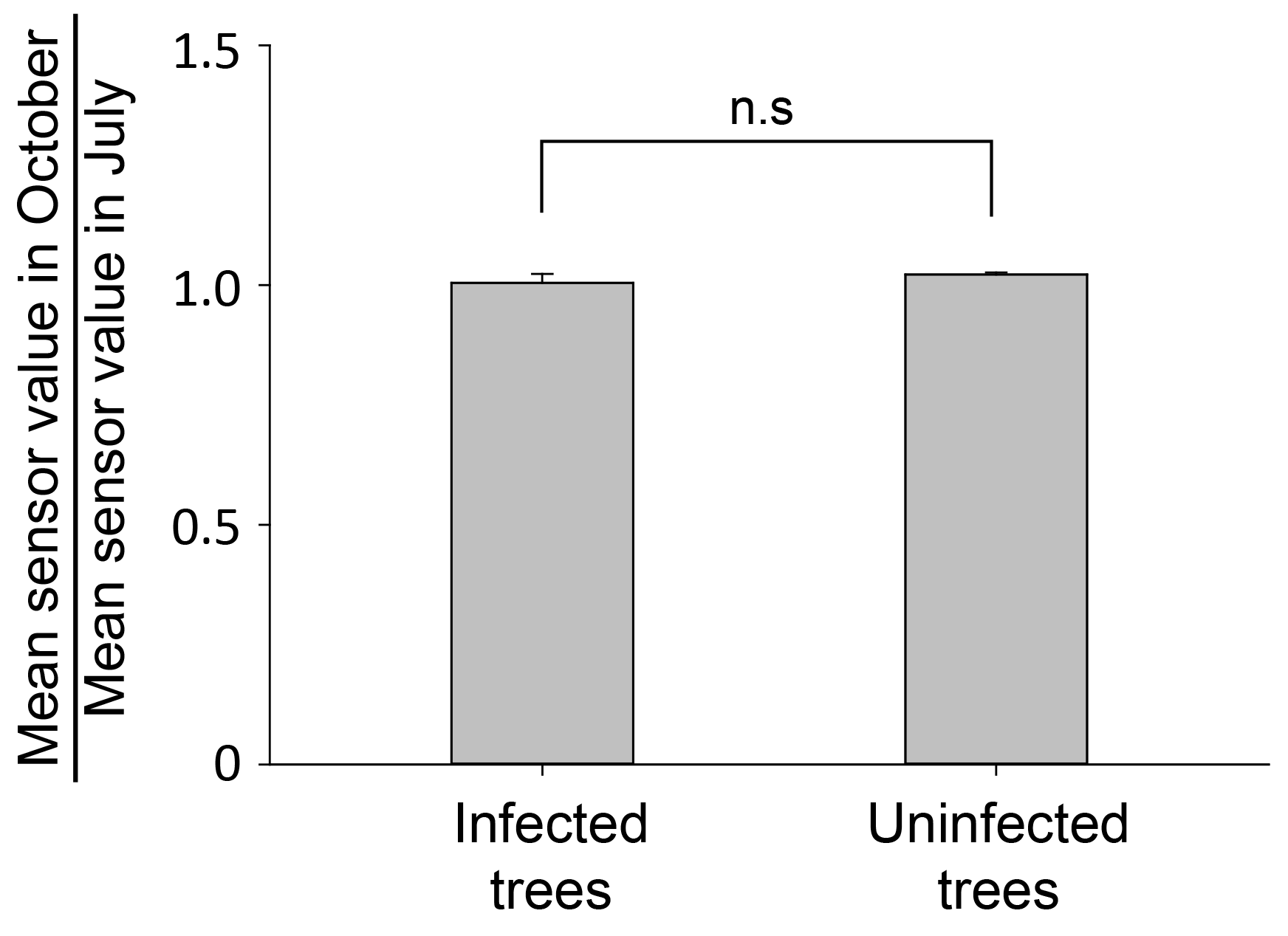

Supplement: S4 Fig — Sample size: n = 14 for infected; n = 51 for uninfected. n.s: not significant (one-way ANOVA on ranks). The bars and error bars stand for means and 95% confidence intervals, respectively. (TIF) [file pone.0257900.s004.tif]
