# Supplementary material for: Diagnosis of pine wilt disease using remote wireless sensing
Source: PLoS One. 2021 Sep 24;16(9):e0257900. doi: 10.1371/journal.pone.0257900 (PMC8462718; doi:10.1371/journal.pone.0257900)
Supplement: S1 File — Photos were taken in January 2020. (PDF) [file pone.0257900.s005.pdf]

S1 File. Photographs of the test trees in Gyeongju for visual observation

Tree #	Photographs taken in January 2020	
Tree #1	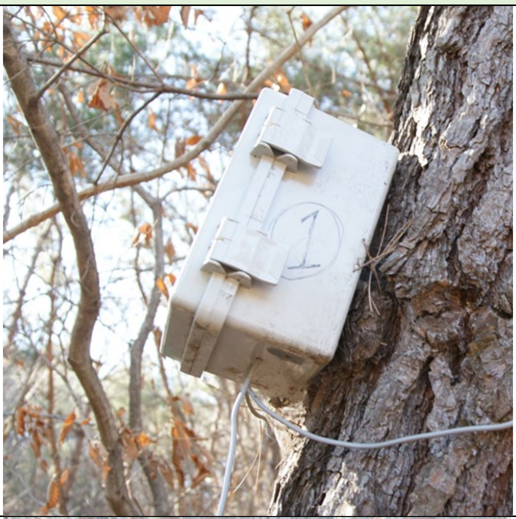	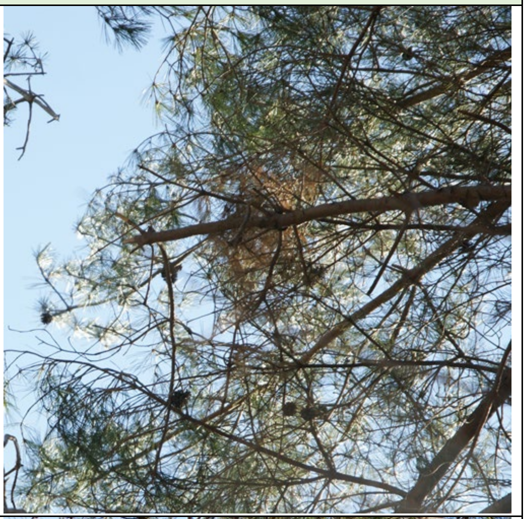
Tree #2	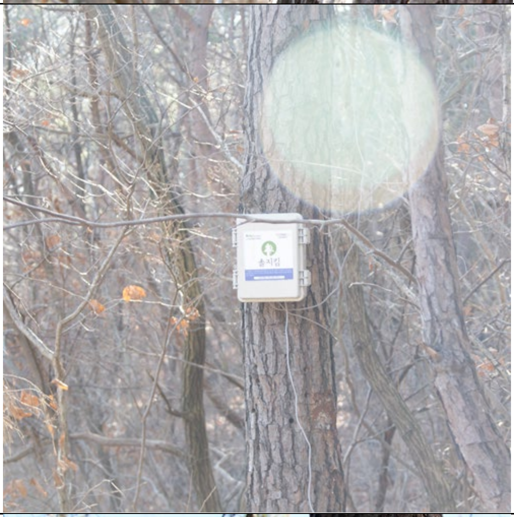	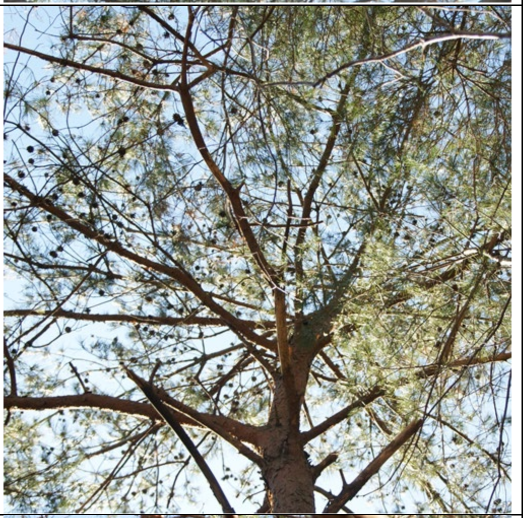
Tree #3	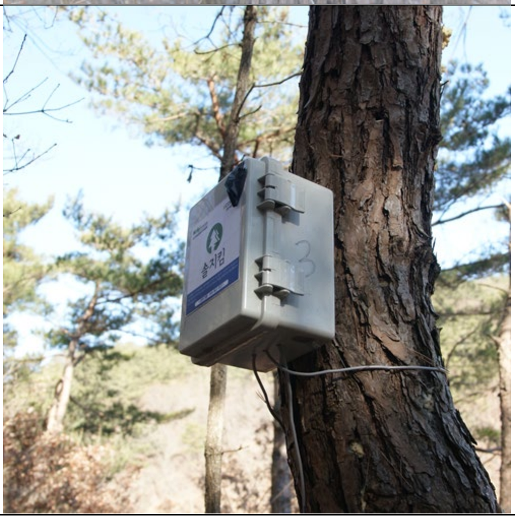	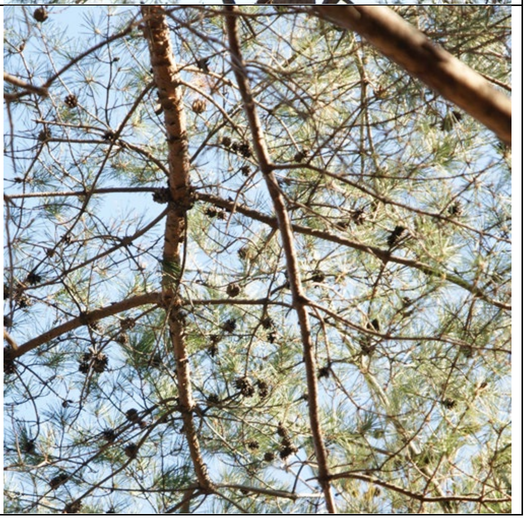

Tree #4	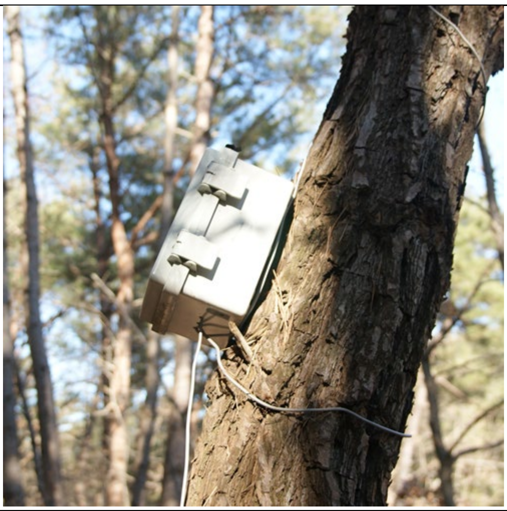	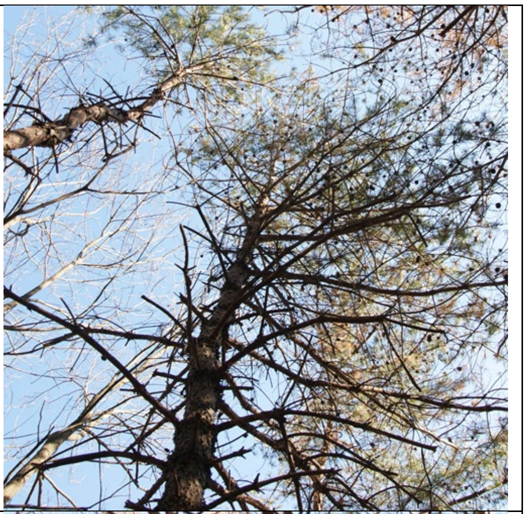
Tree #5	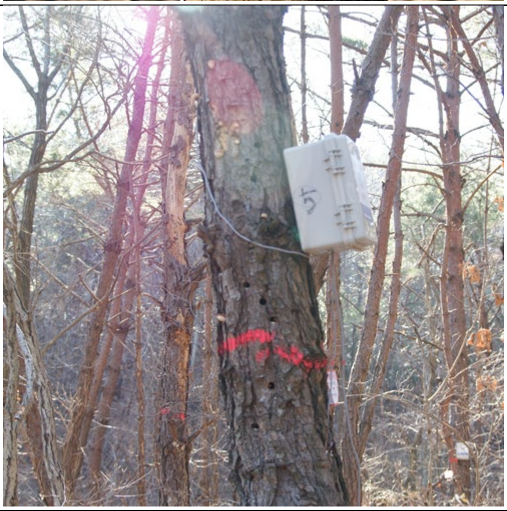	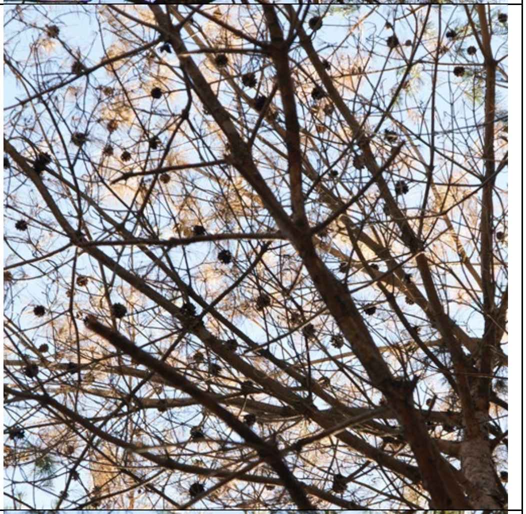
Tree #6	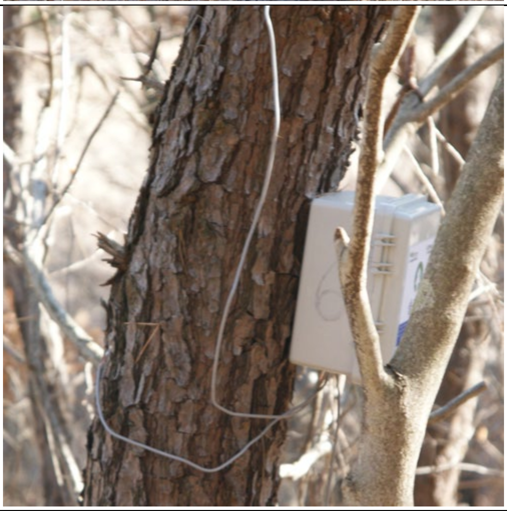	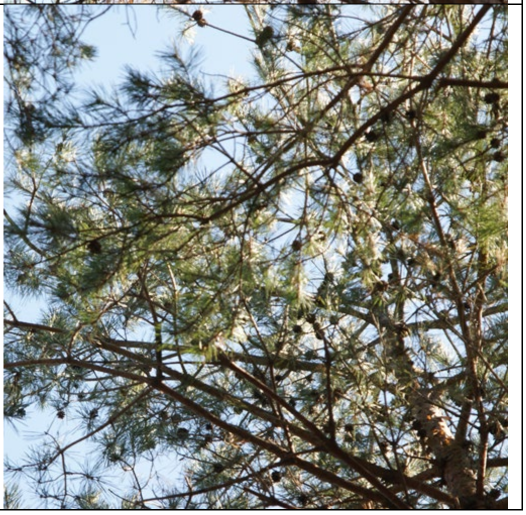

Tree #7	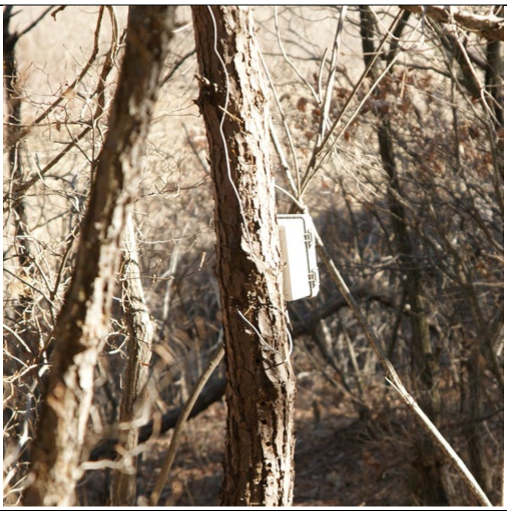	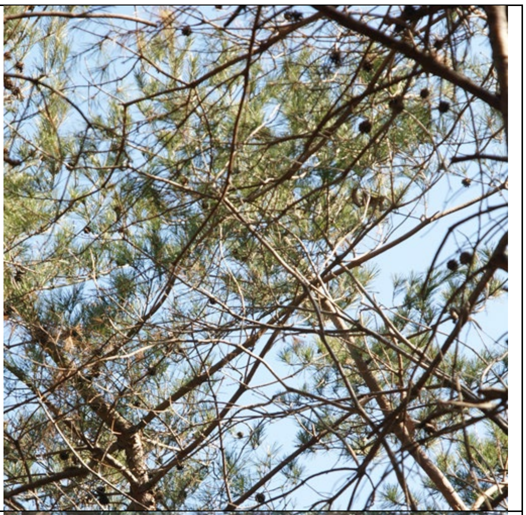
Tree #8	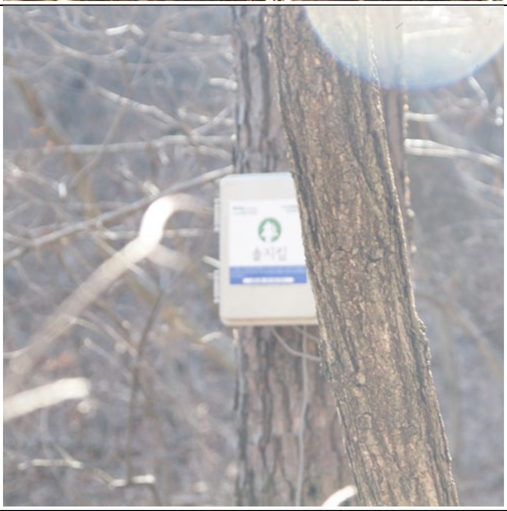	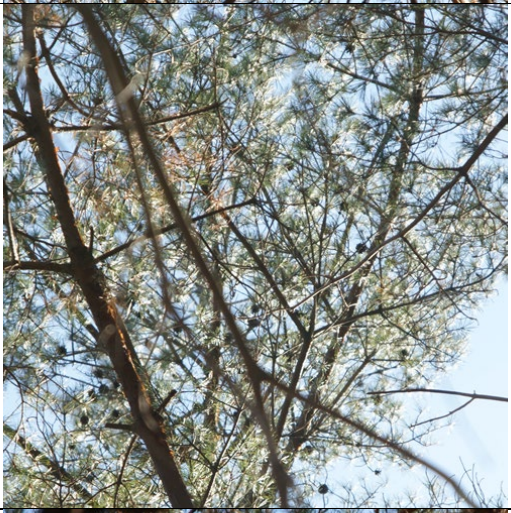
Tree #9	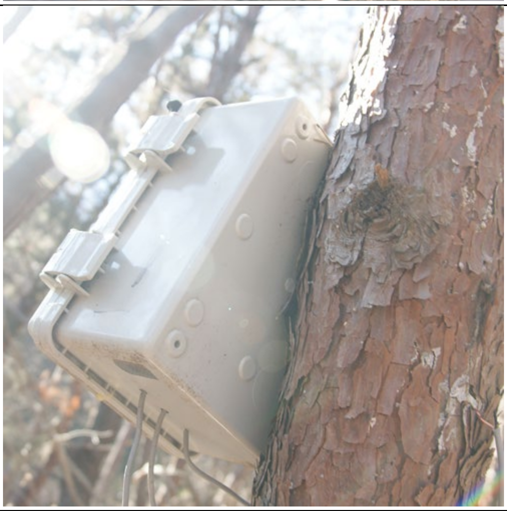	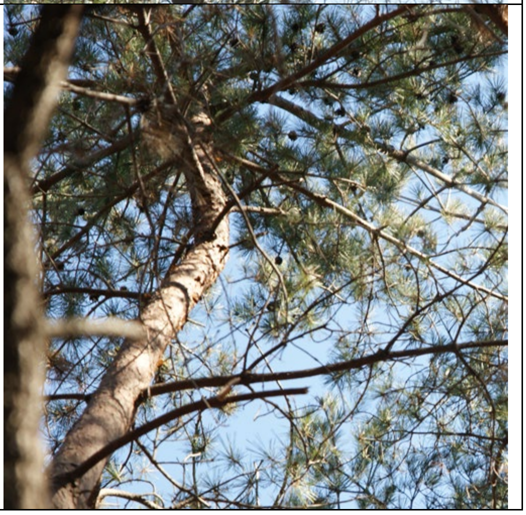

Tree #10	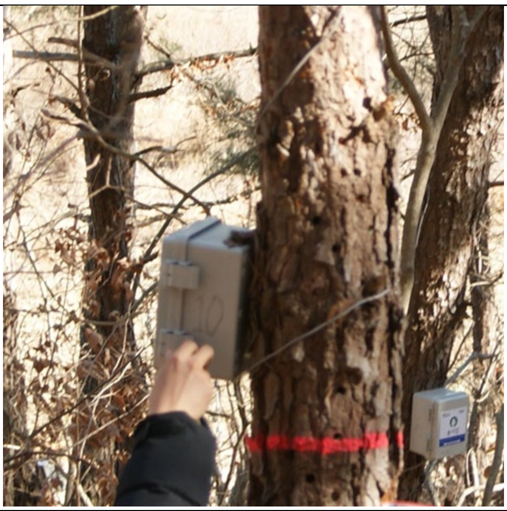	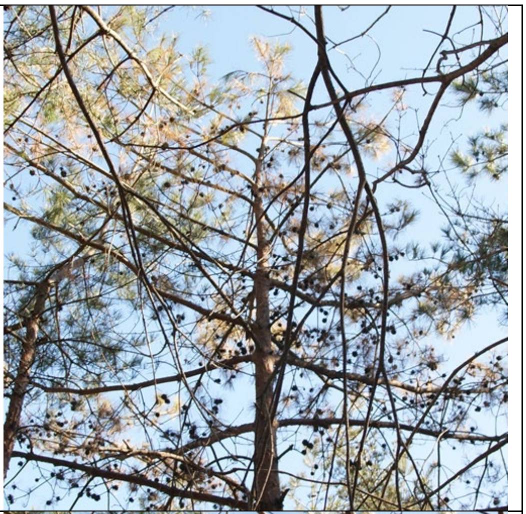
Tree #11	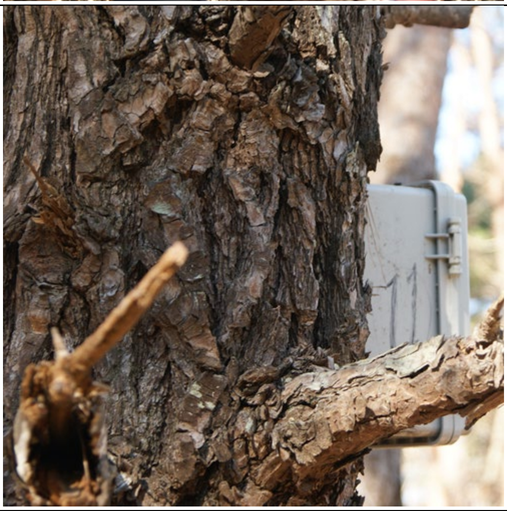	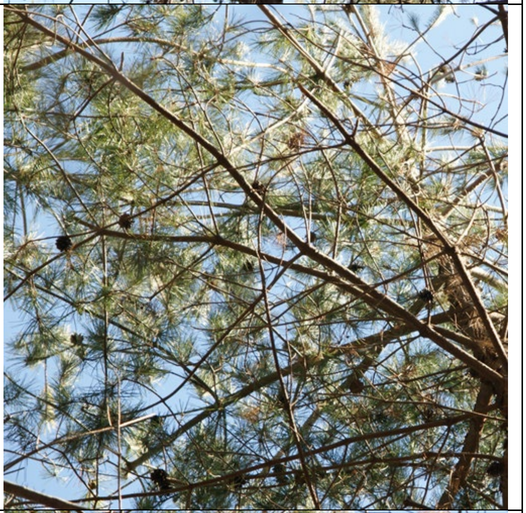
Tree #12	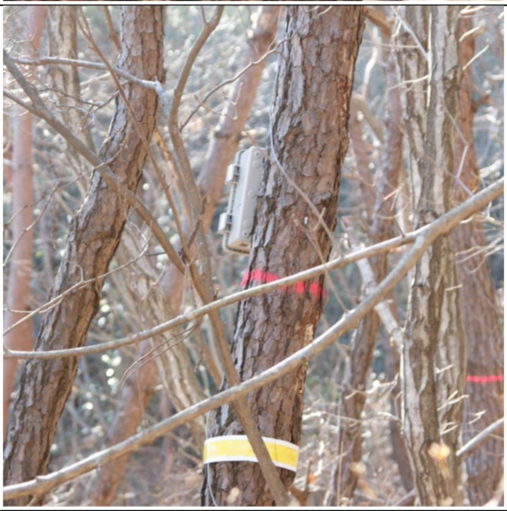	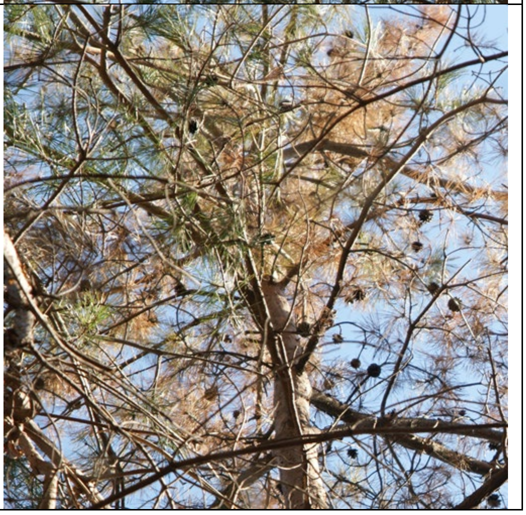

Tree #13	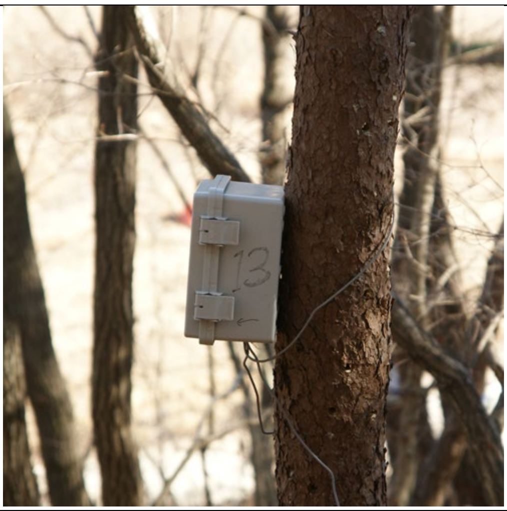	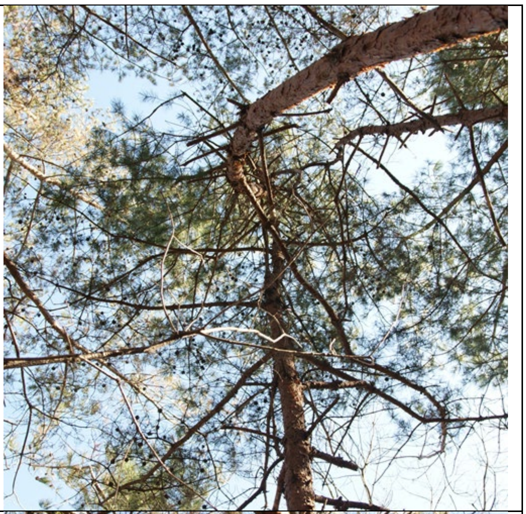
Tree #14	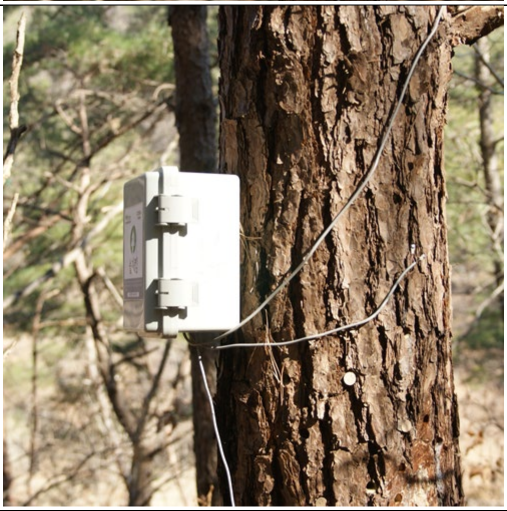	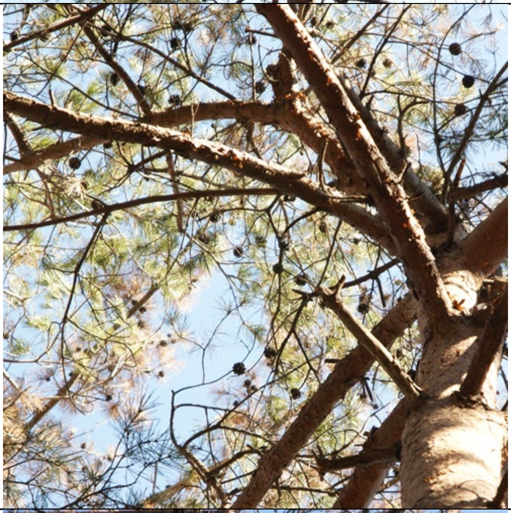
Tree #15	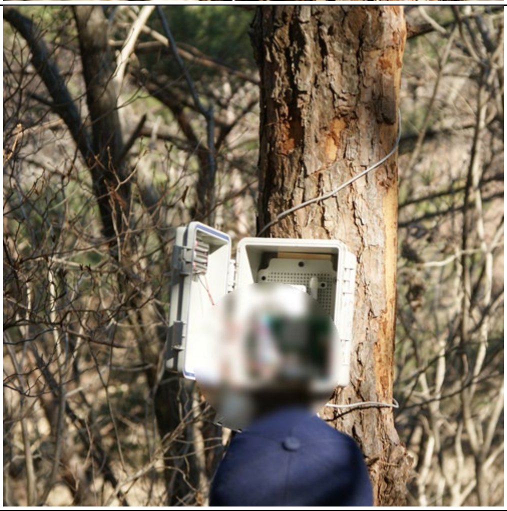	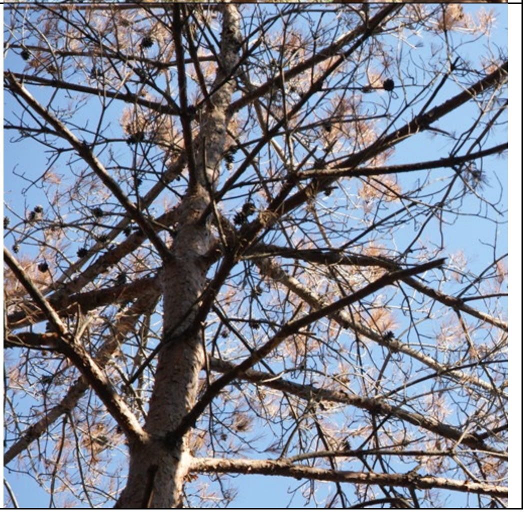

Tree #16	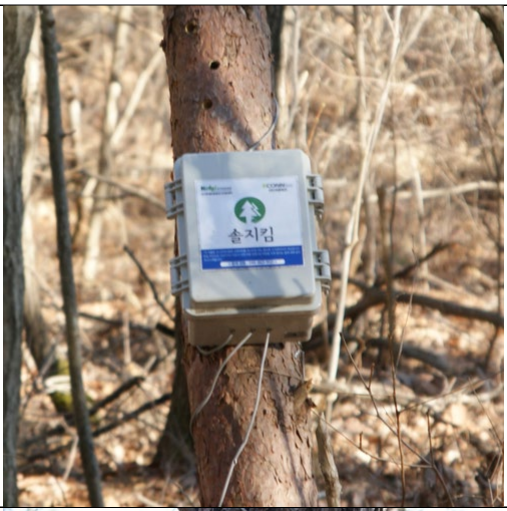	
Tree #17	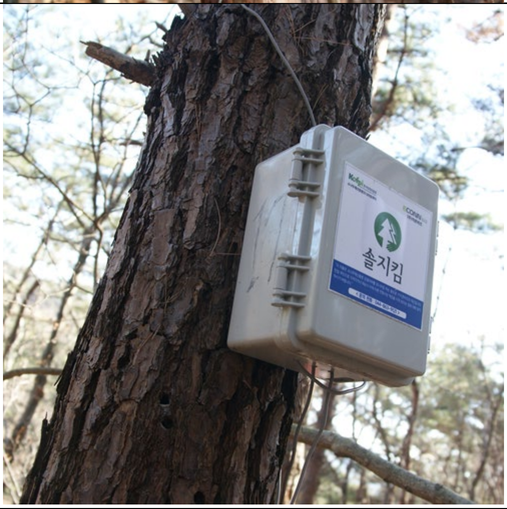	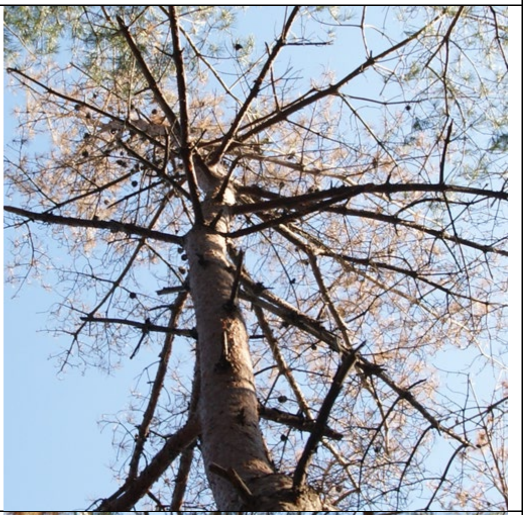
Tree #18	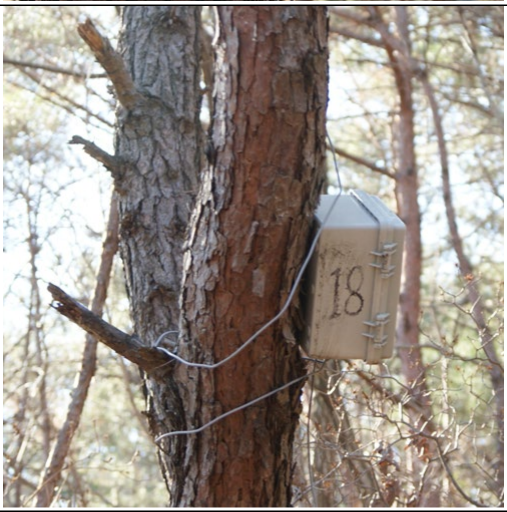	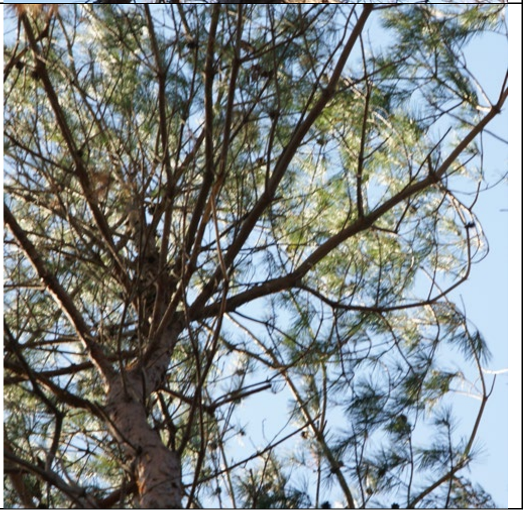

Tree #19	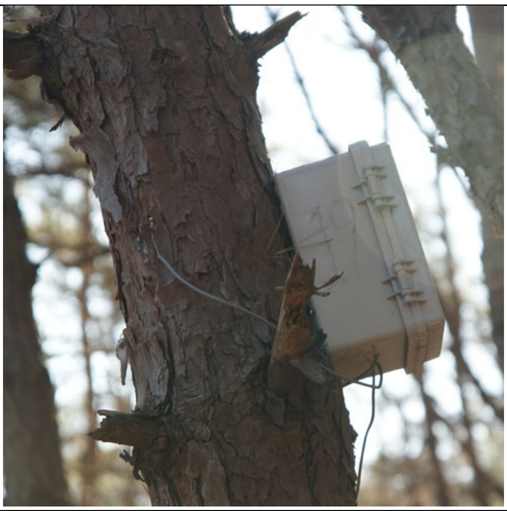	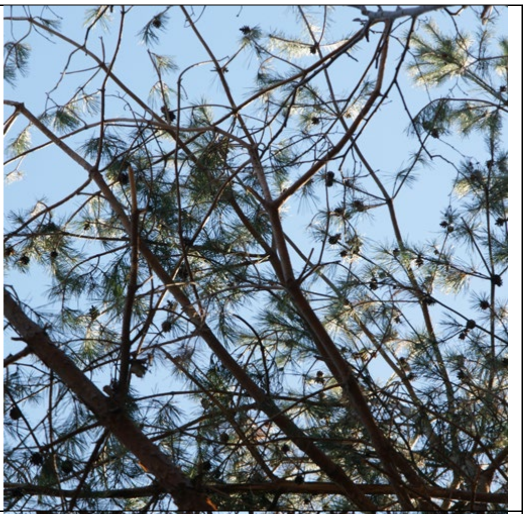
Tree #20	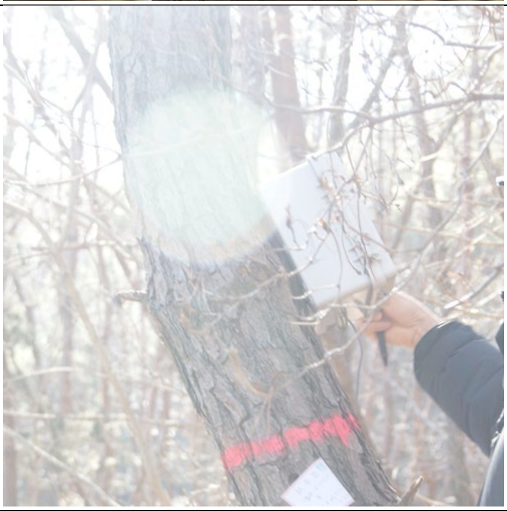	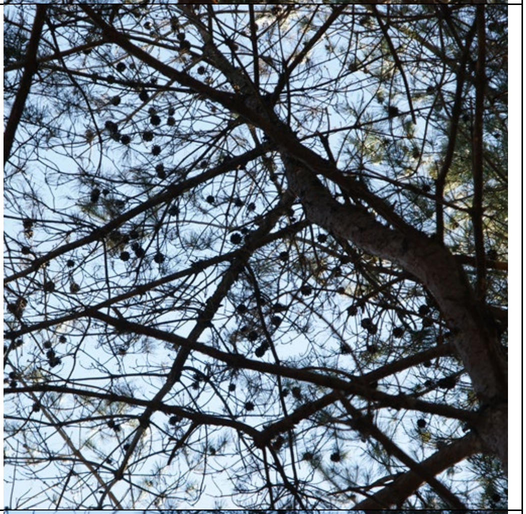
Tree #21	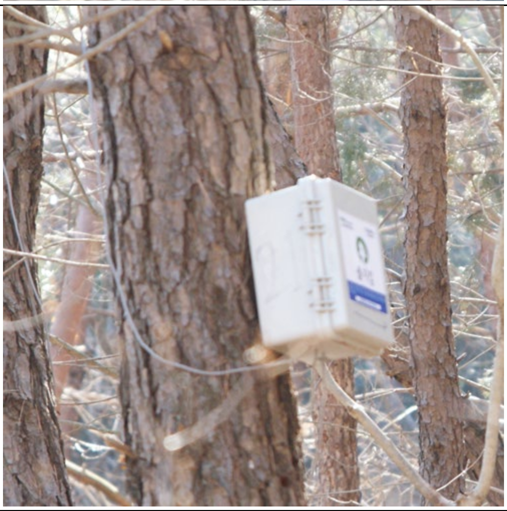	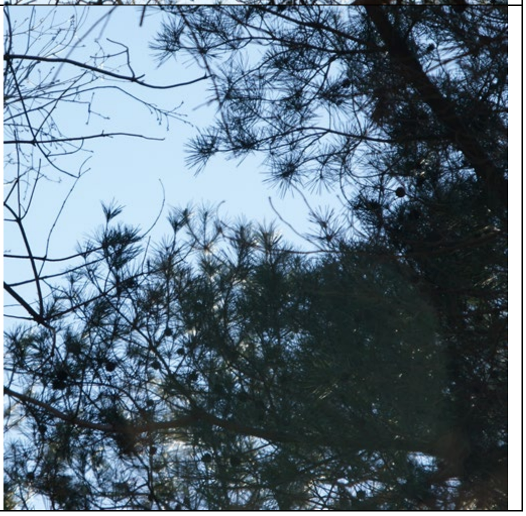

Tree #22	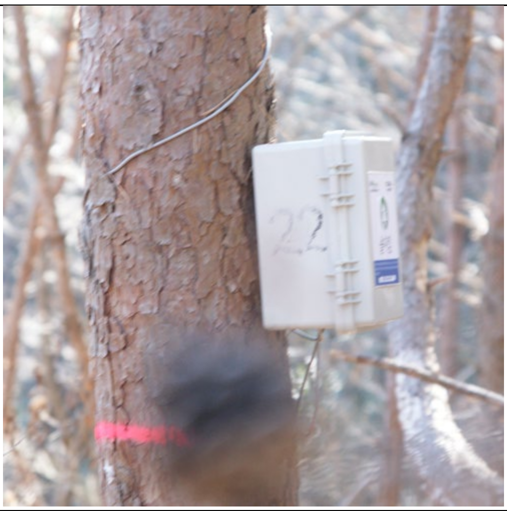	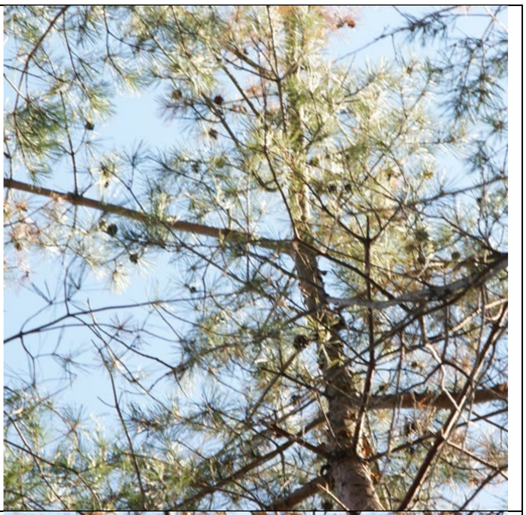
Tree #23	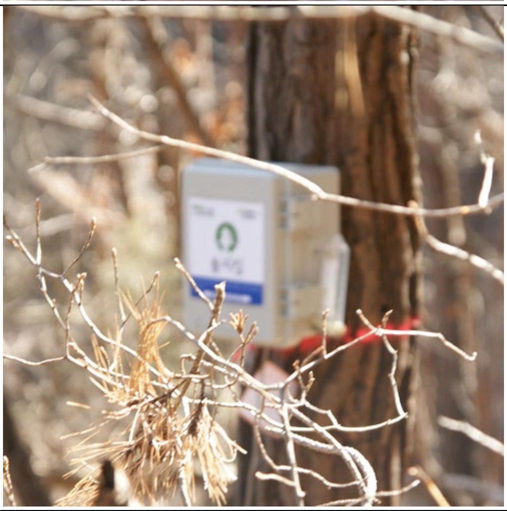	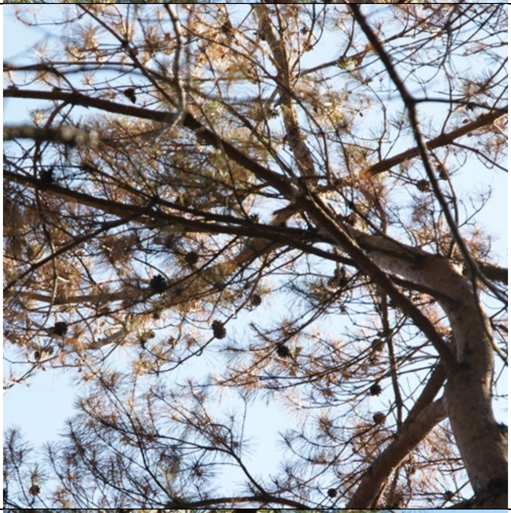
Tree #24	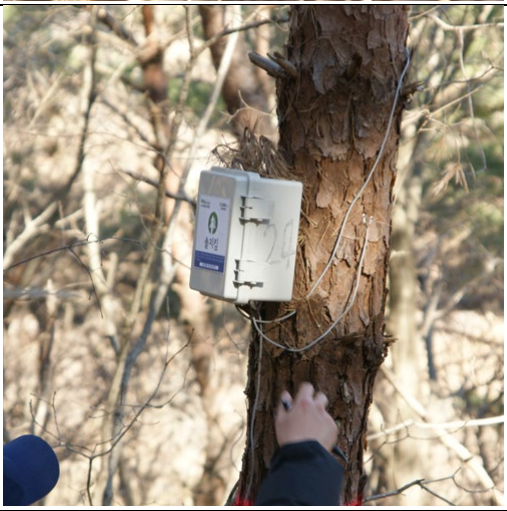	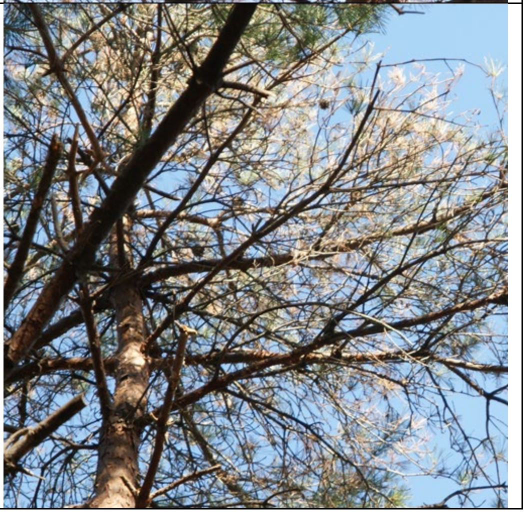

Tree #26	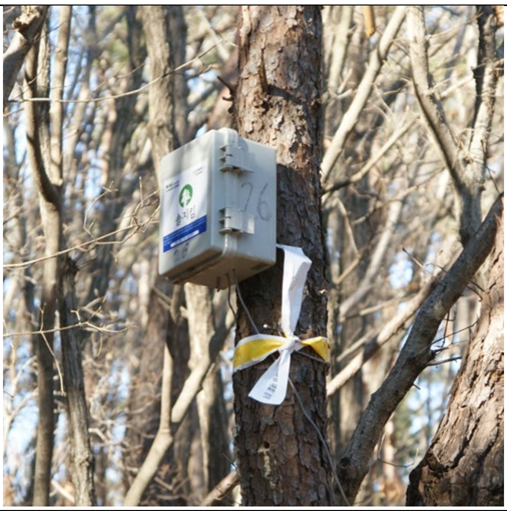	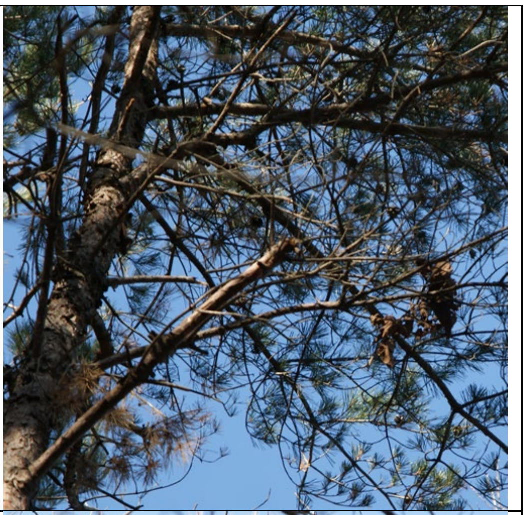
Tree #27	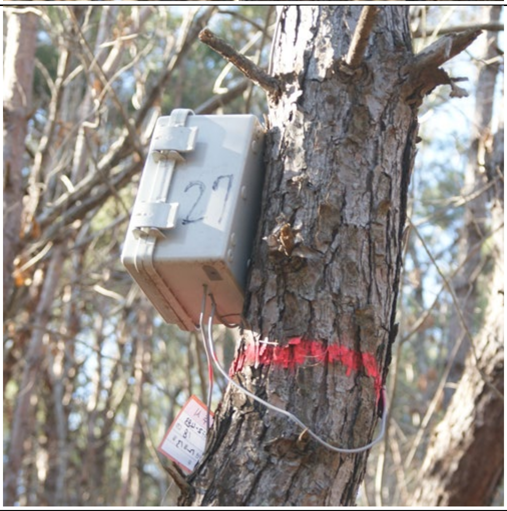	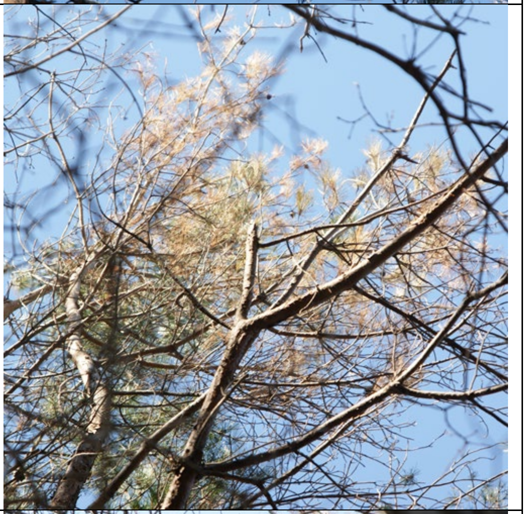
Tree #28	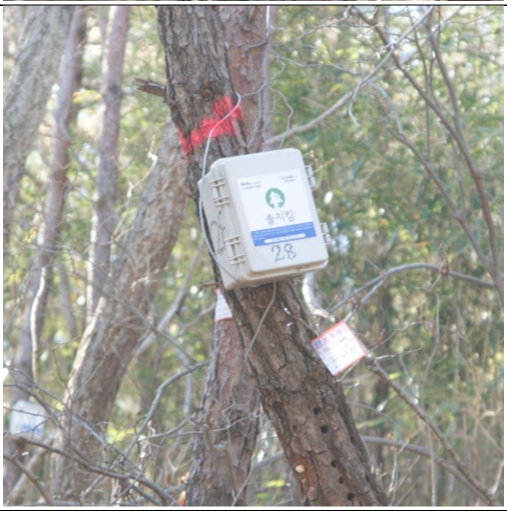	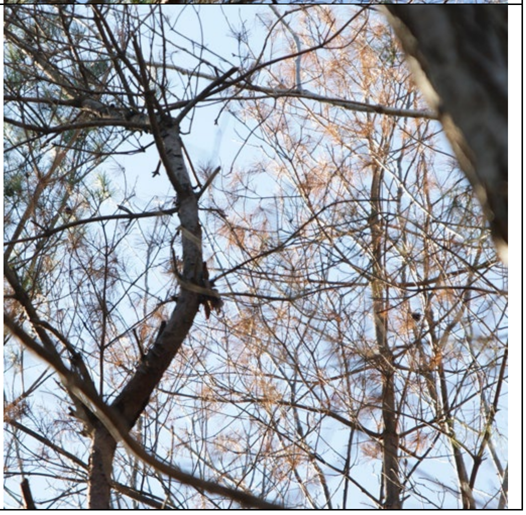

Tree #29	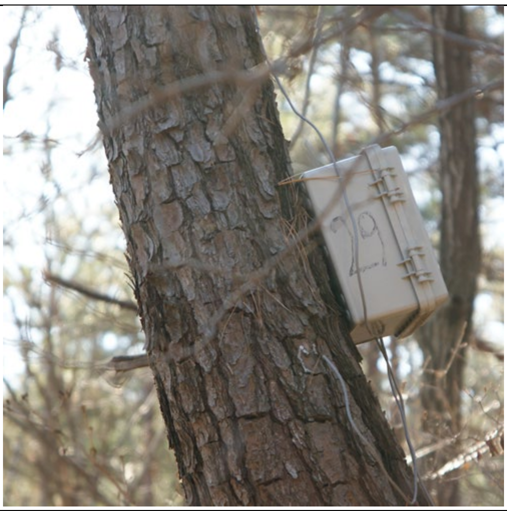	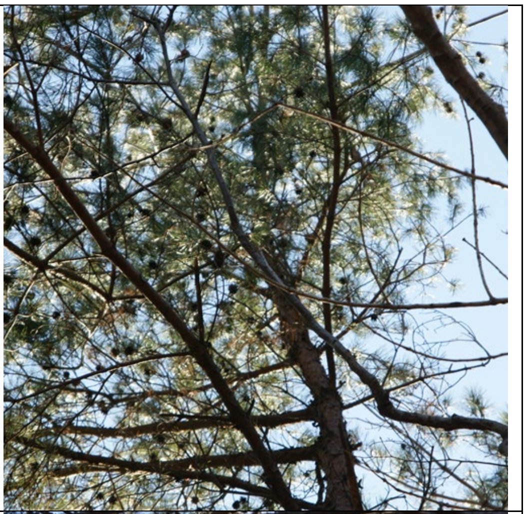
Tree #30	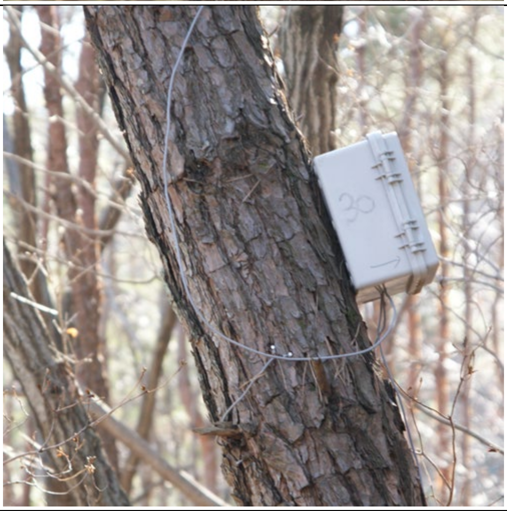	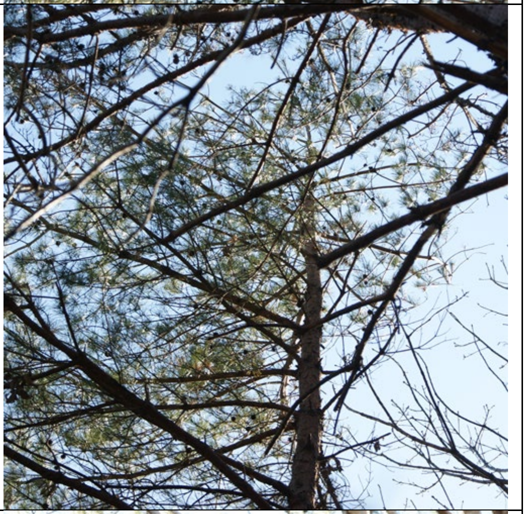
Tree #31	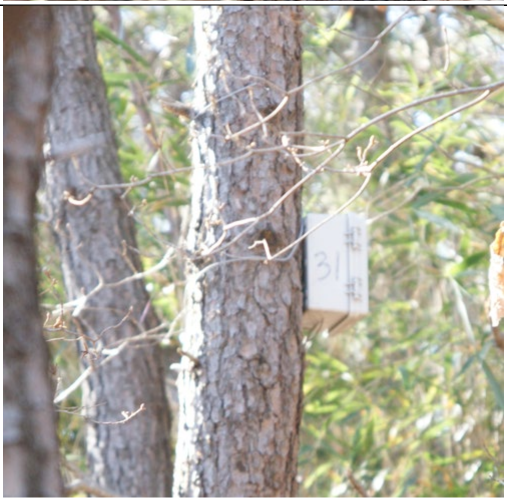	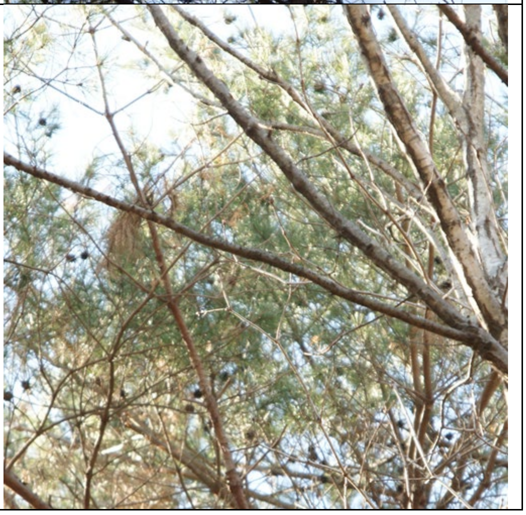

Tree #32	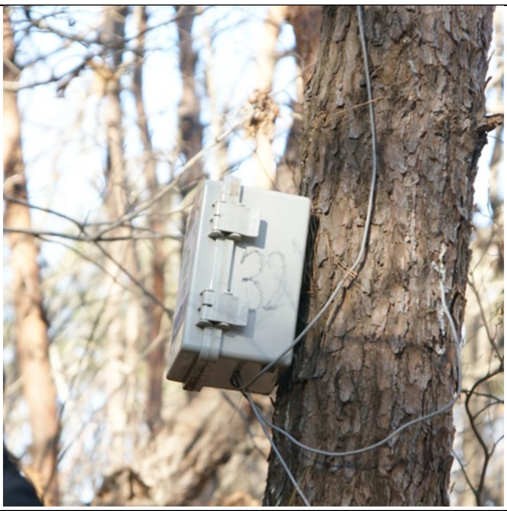	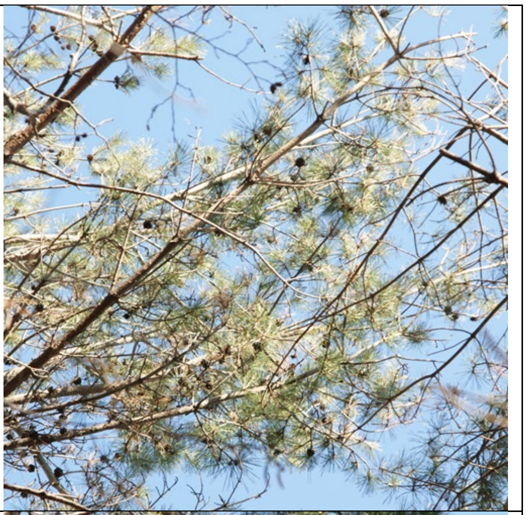
Tree #33	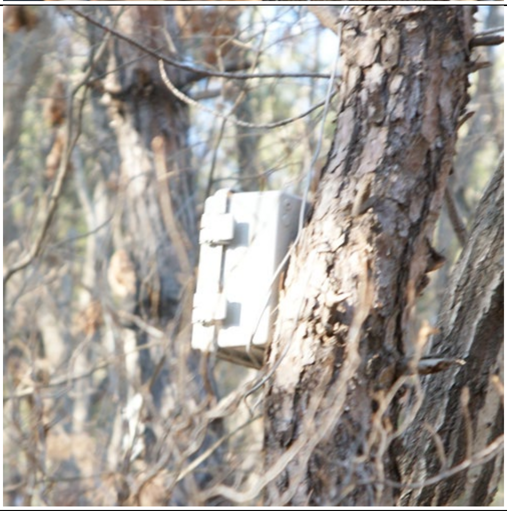	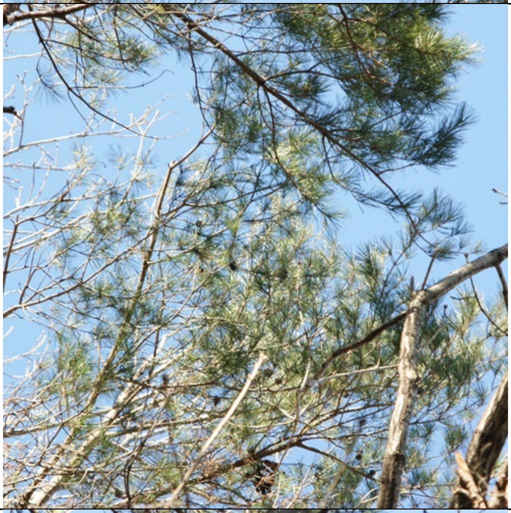
Tree #34	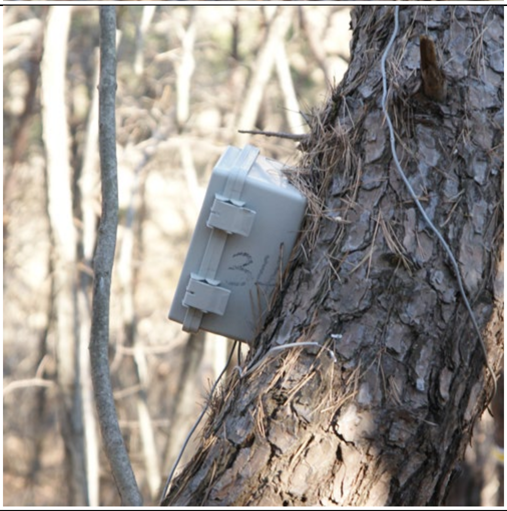	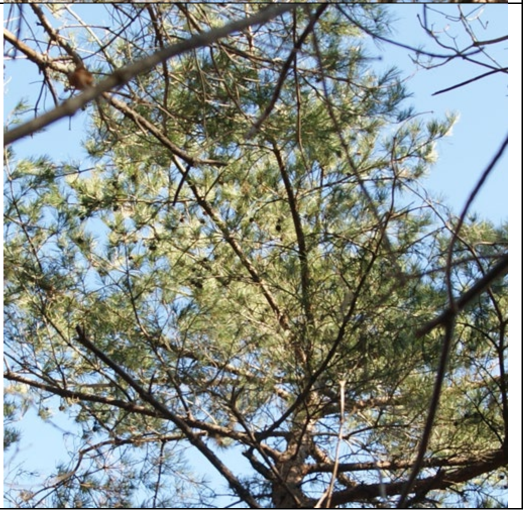

Tree #35	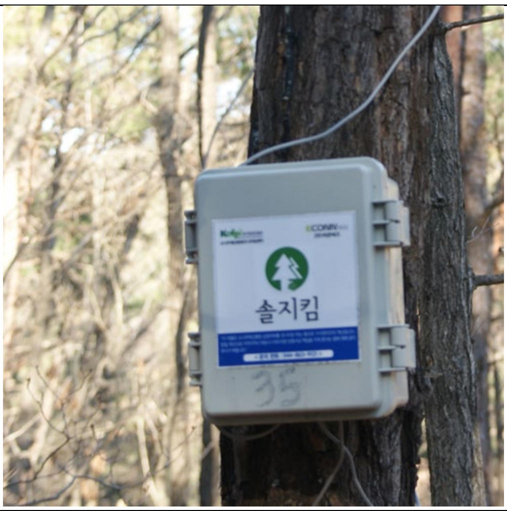	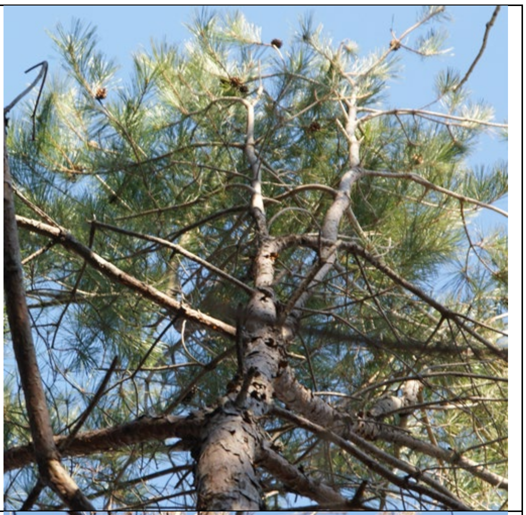
Tree #36	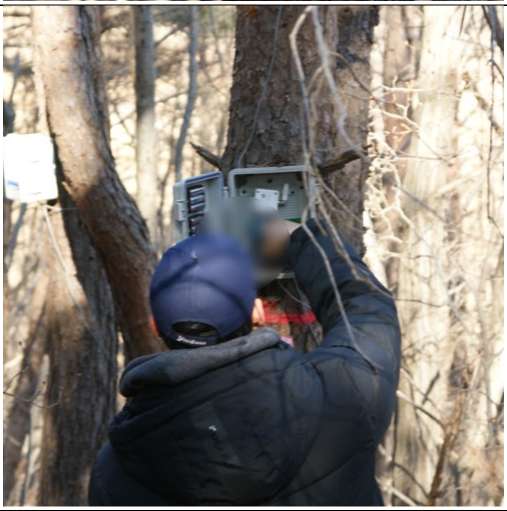	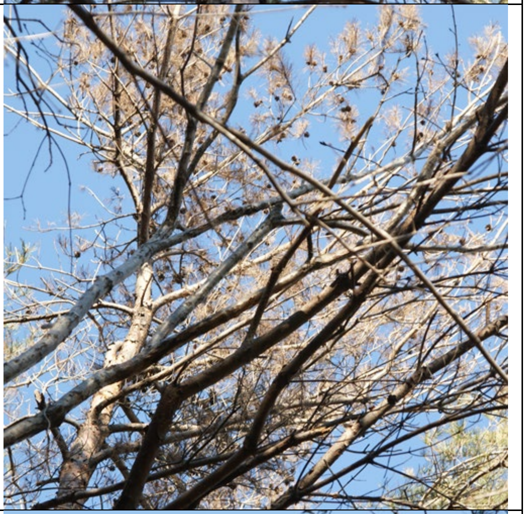
Tree #37	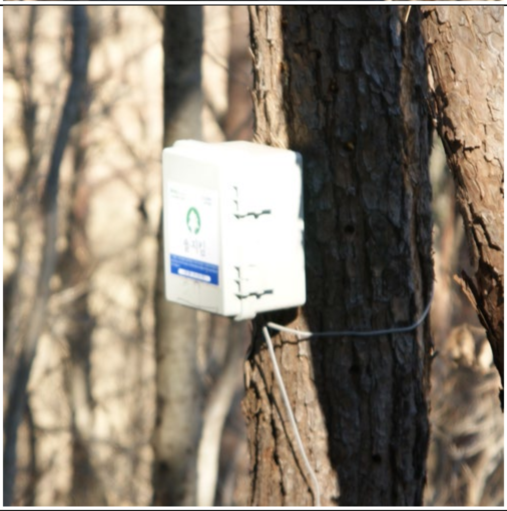	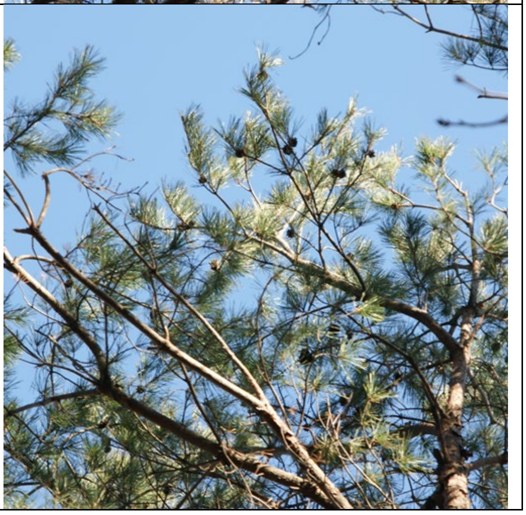

Tree #38	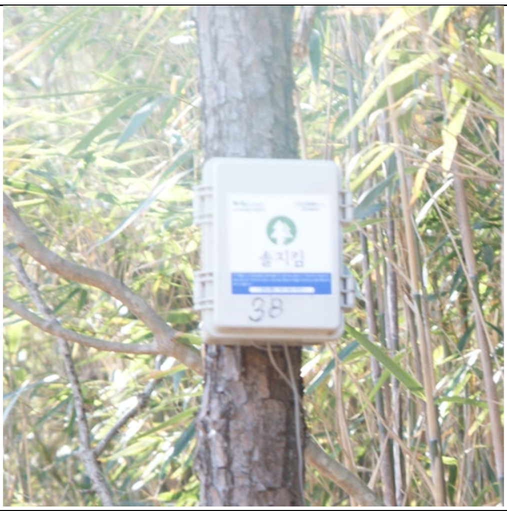	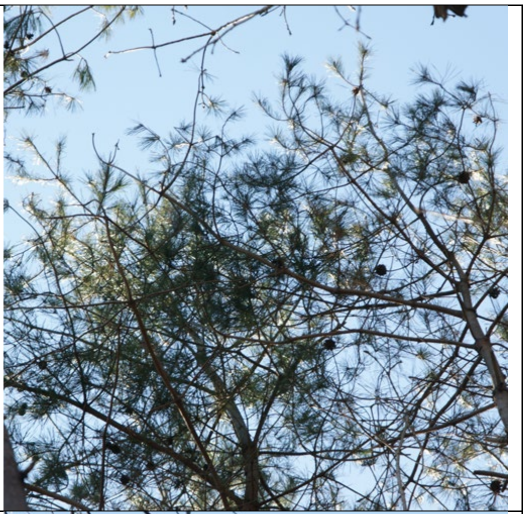
Tree #39	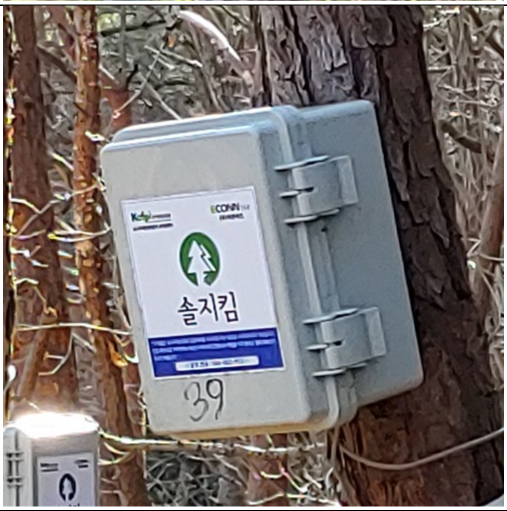	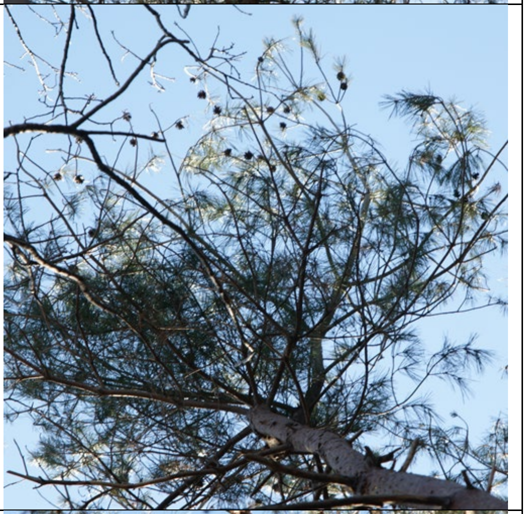
Tree #40	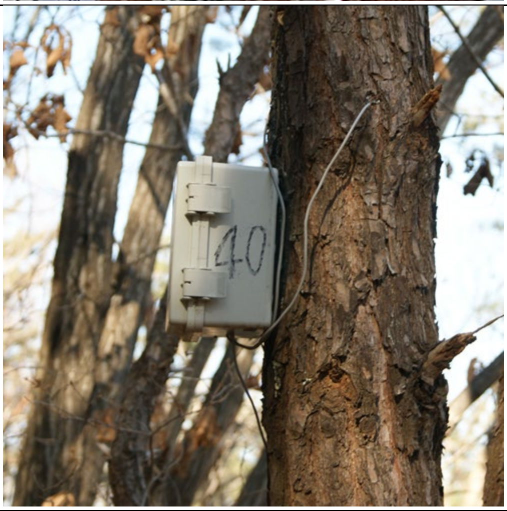	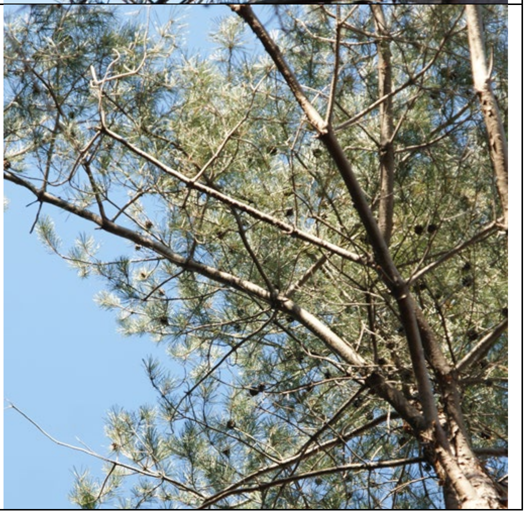

Tree #42	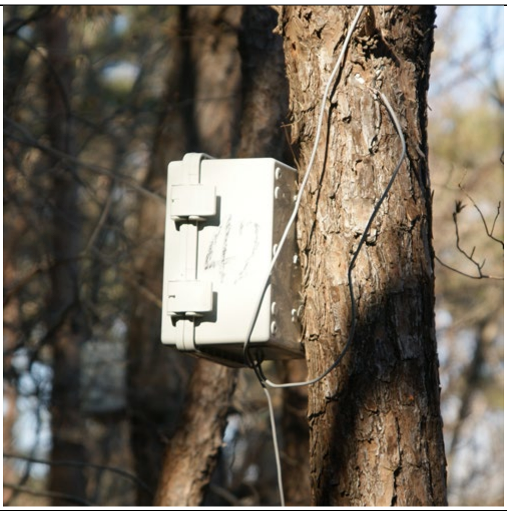	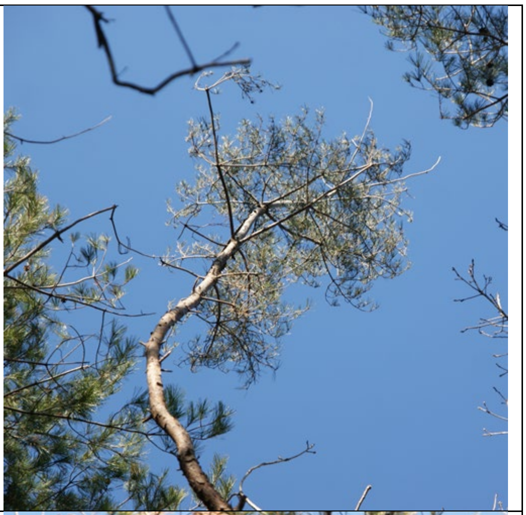
Tree #43	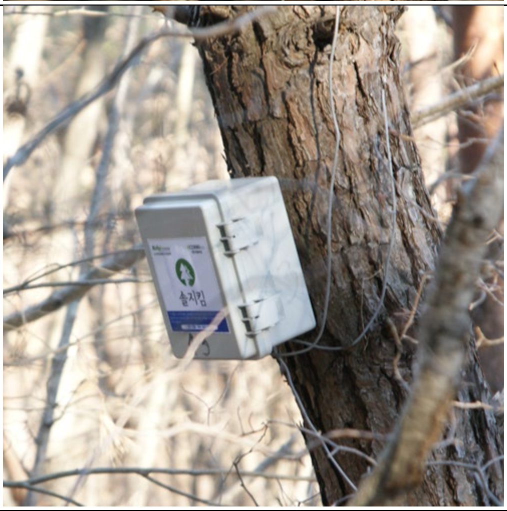	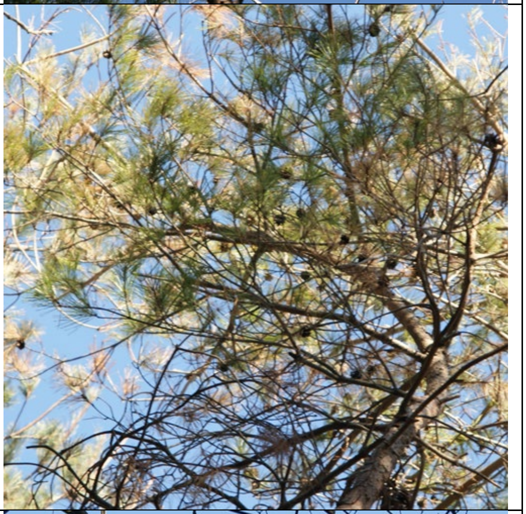
Tree #45	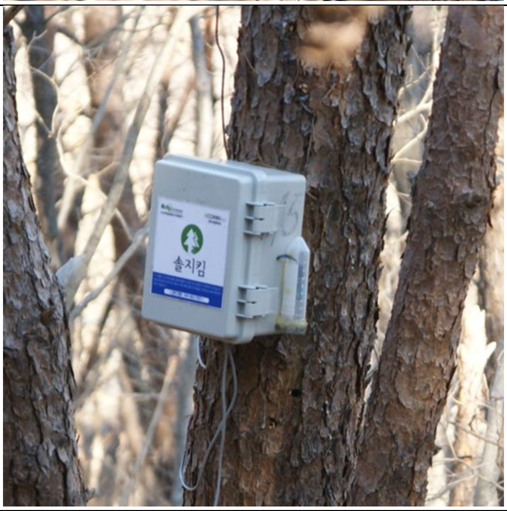	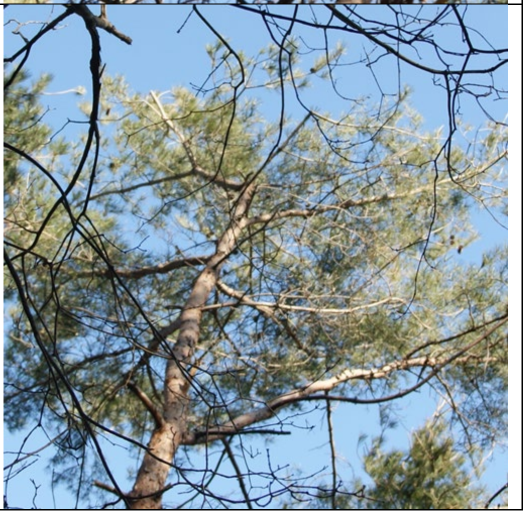

Tree #47	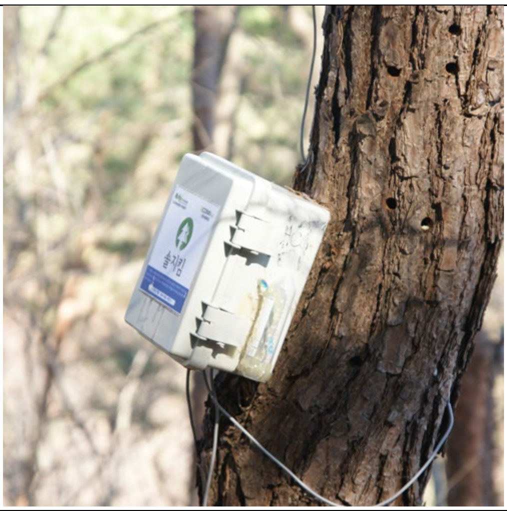	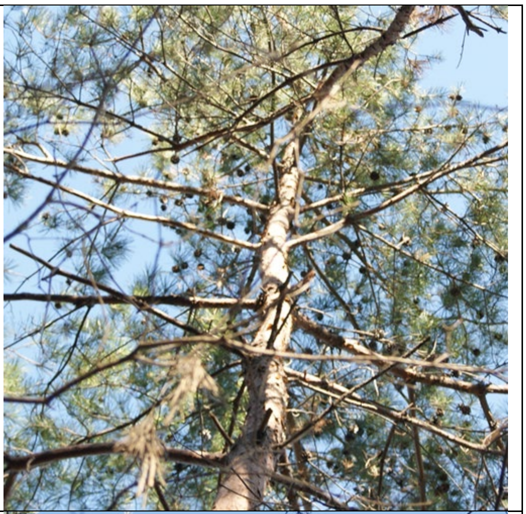
Tree #48	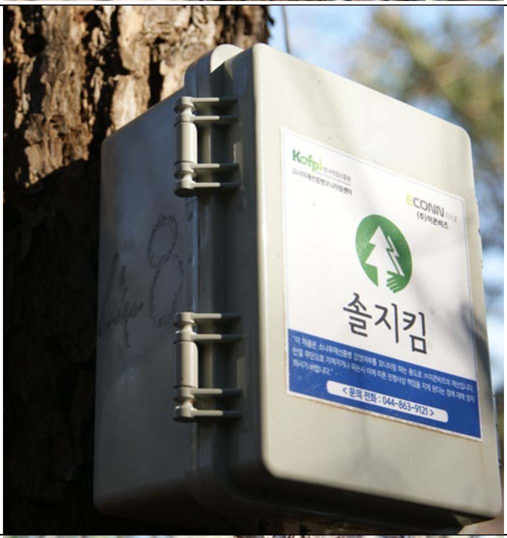	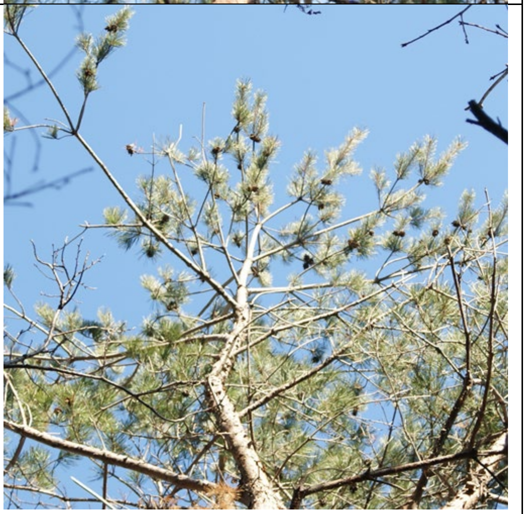
Tree #49	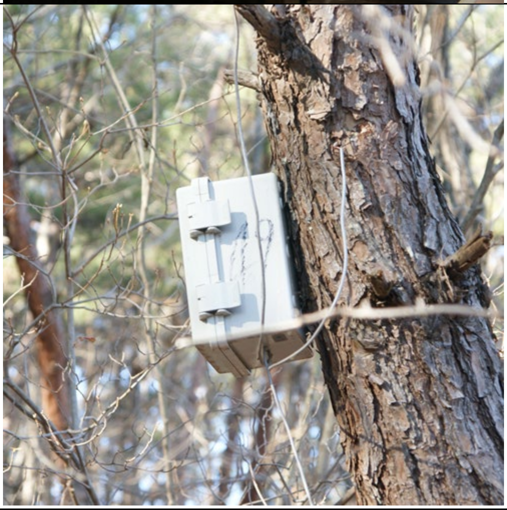	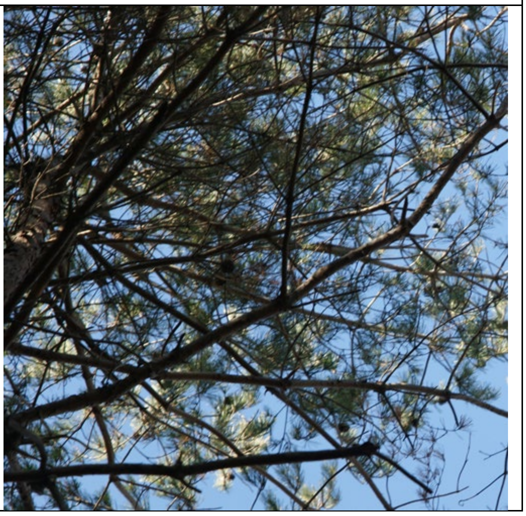

Tree #50	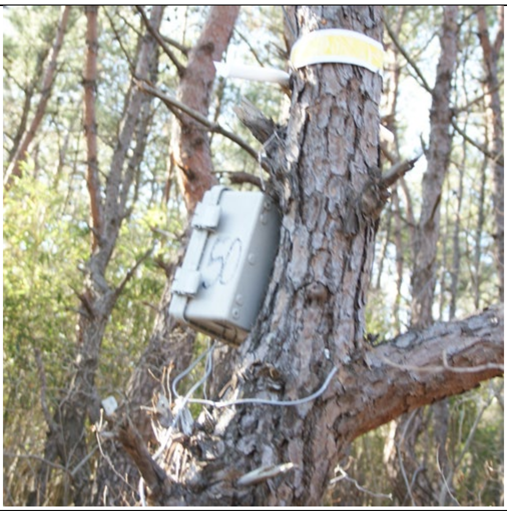	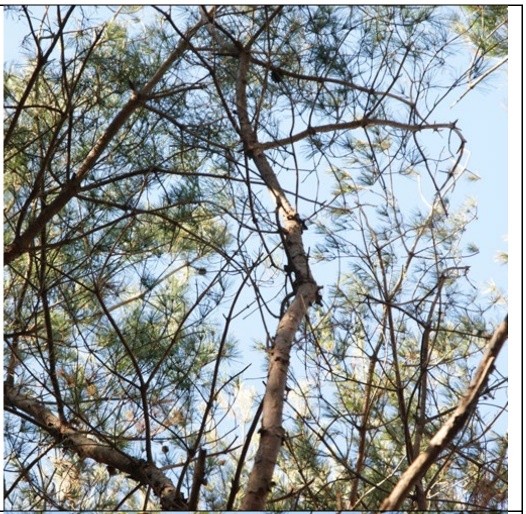
Tree #51	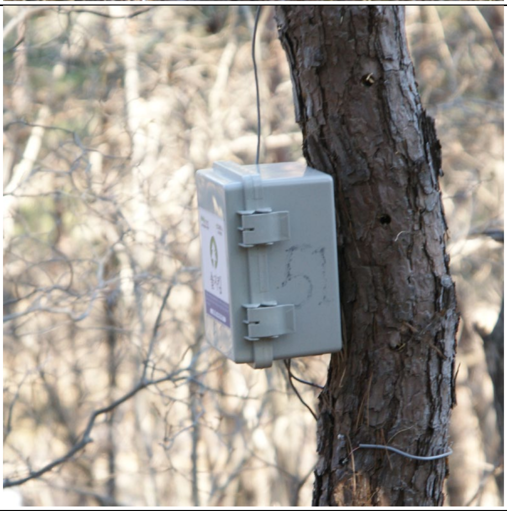	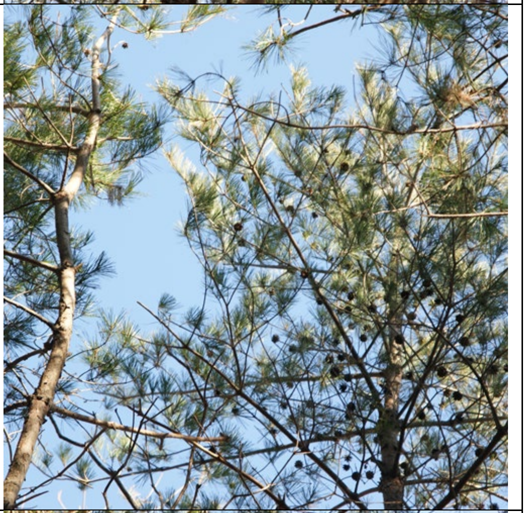
Tree #53	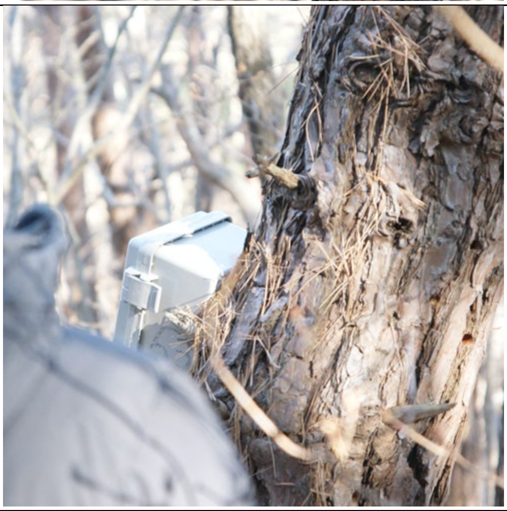	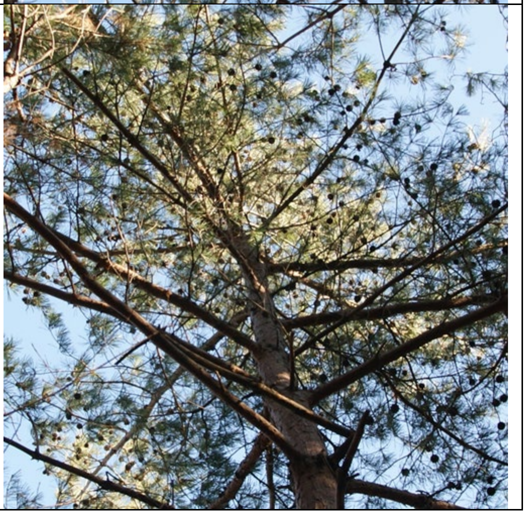

Tree #54	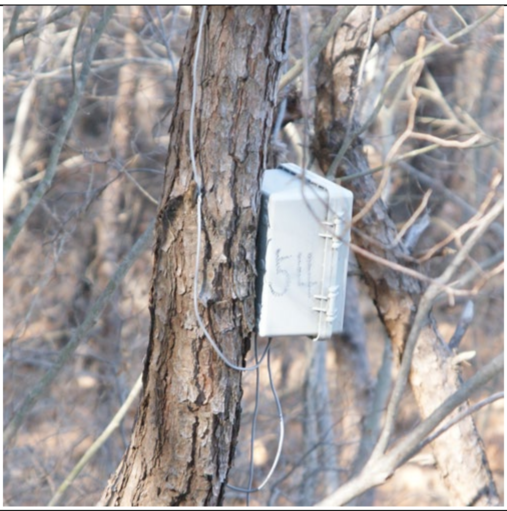	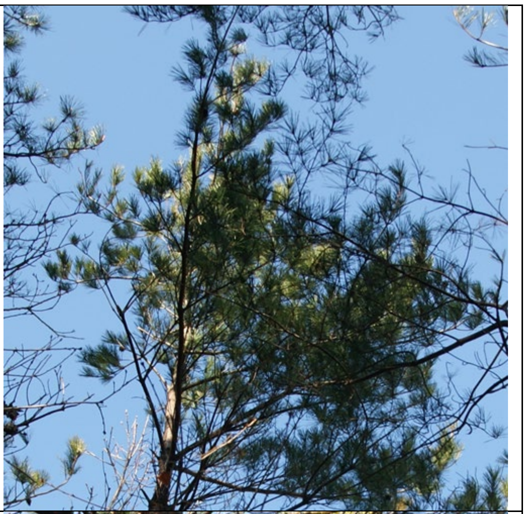
Tree #55	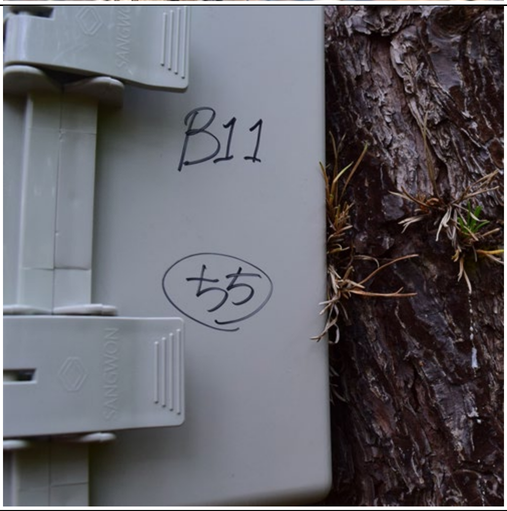	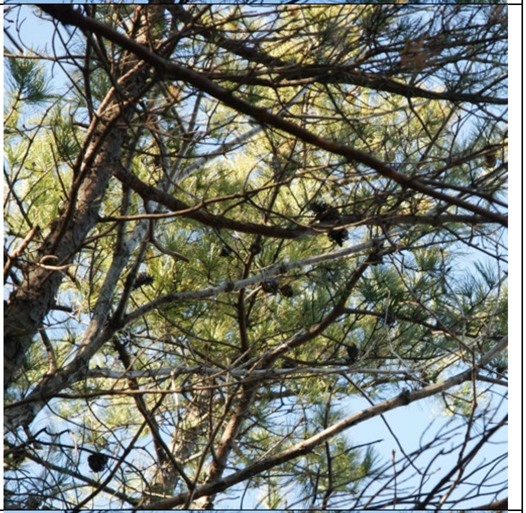
Tree #56	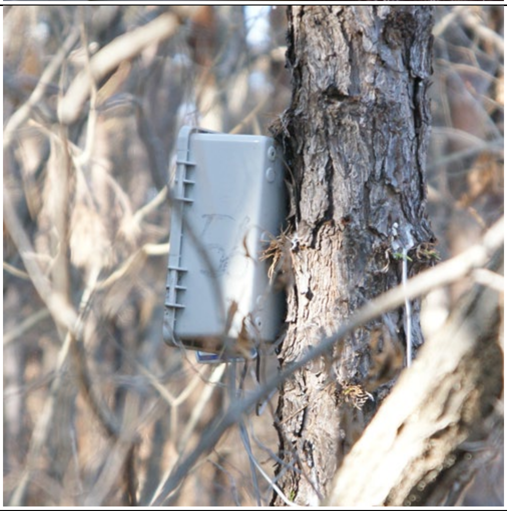	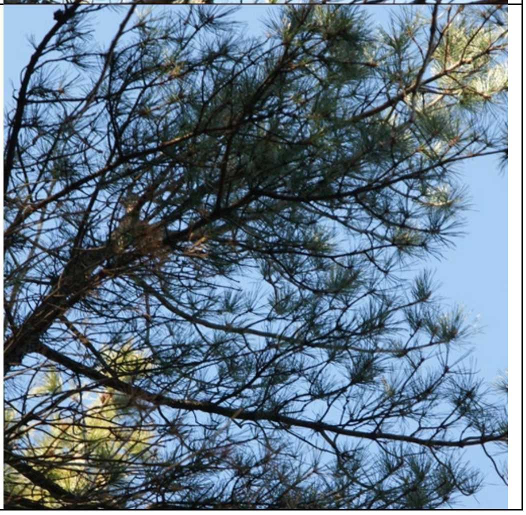

Tree #57	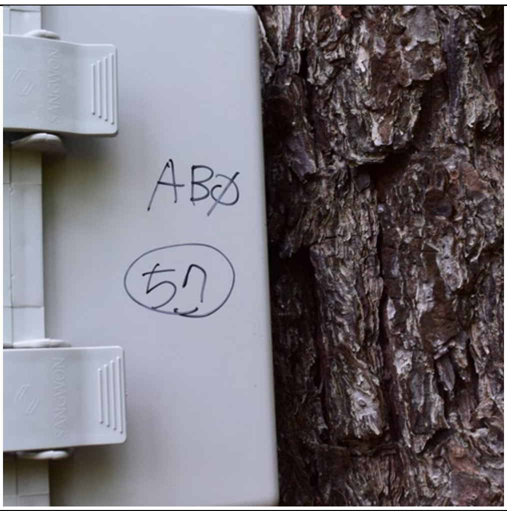	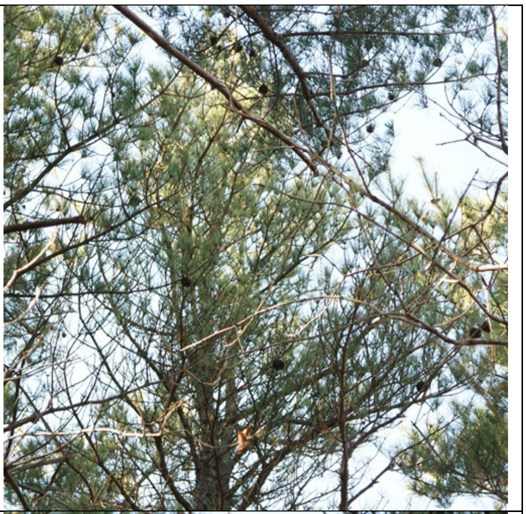
Tree #58	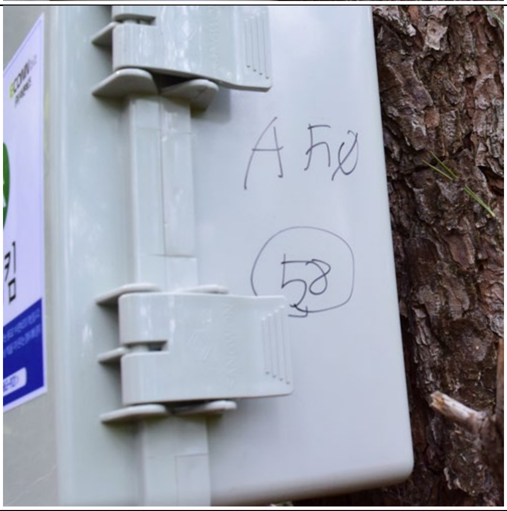	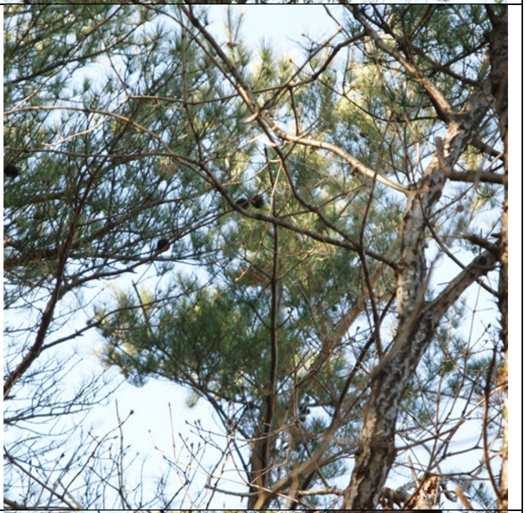
Tree #59	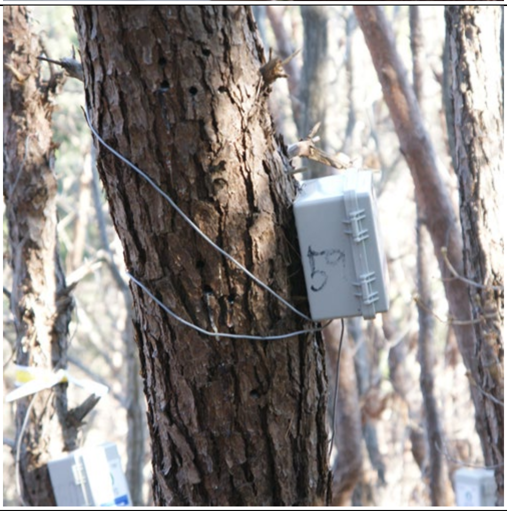	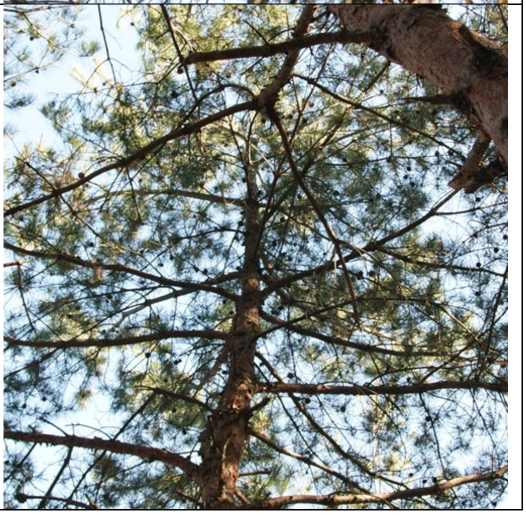

Tree #60	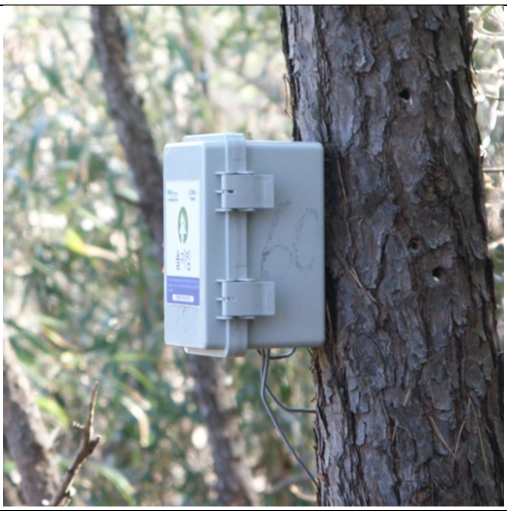	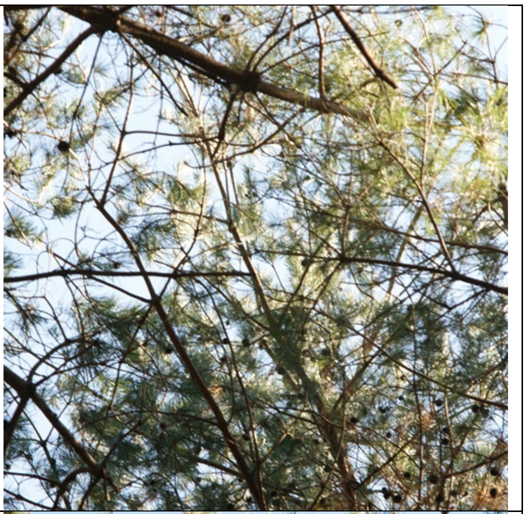
Tree #61	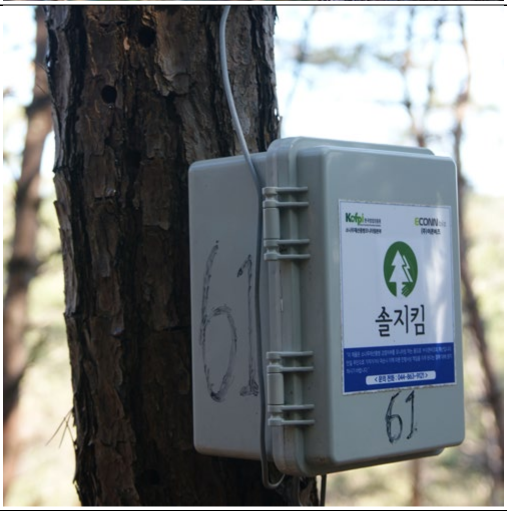	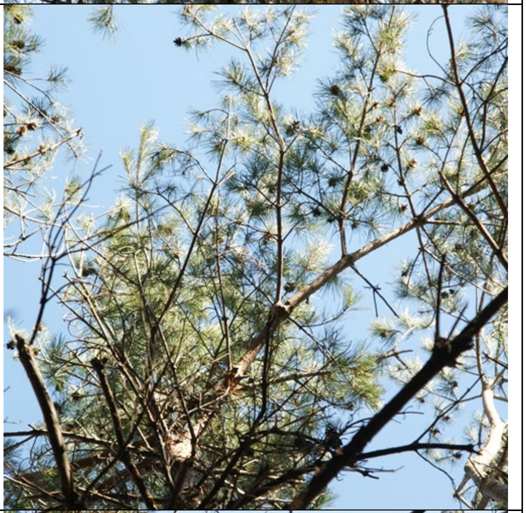
Tree #62	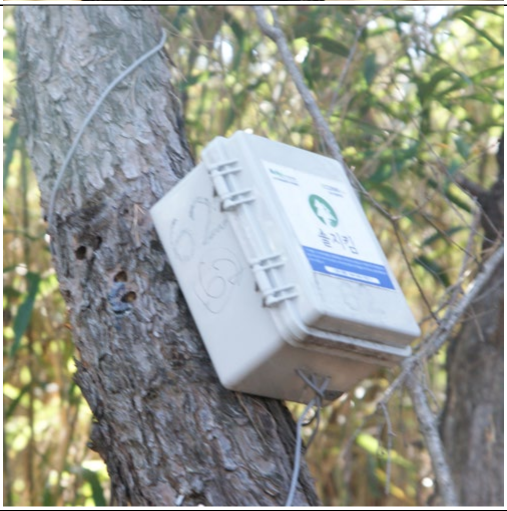	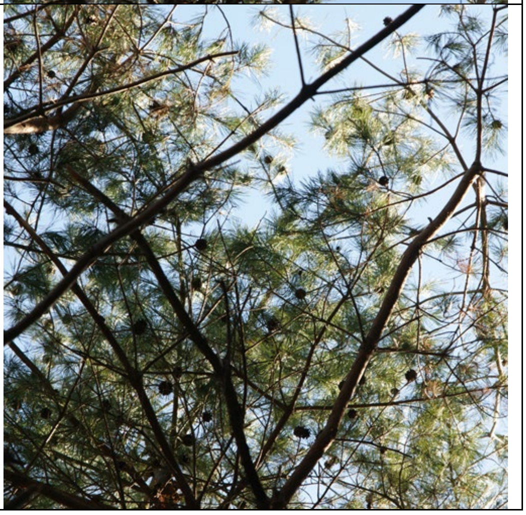

Tree #63	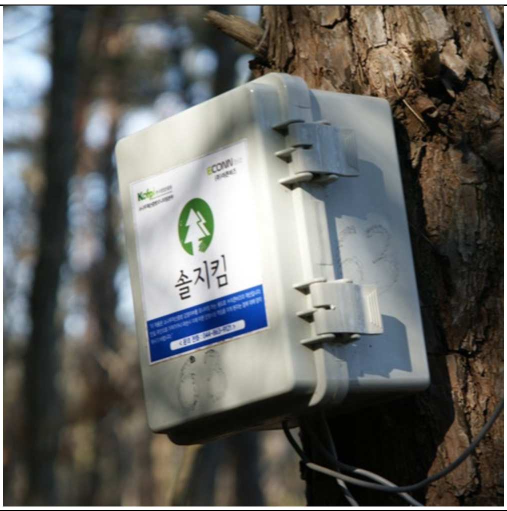	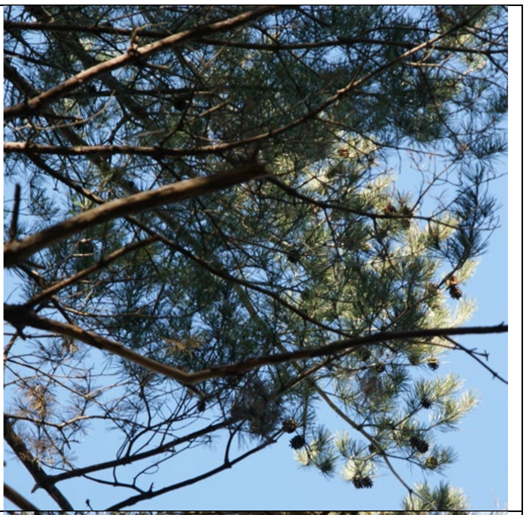
Tree #64	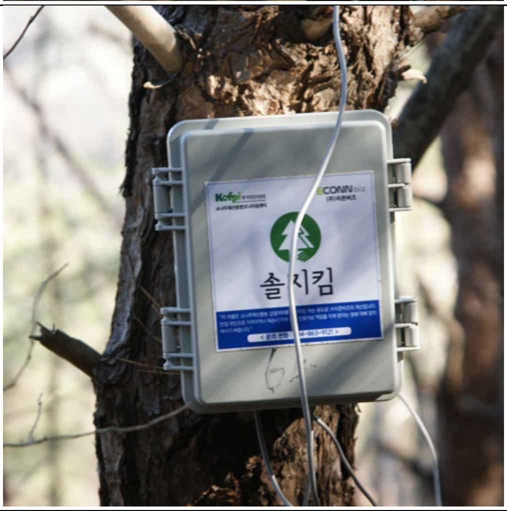	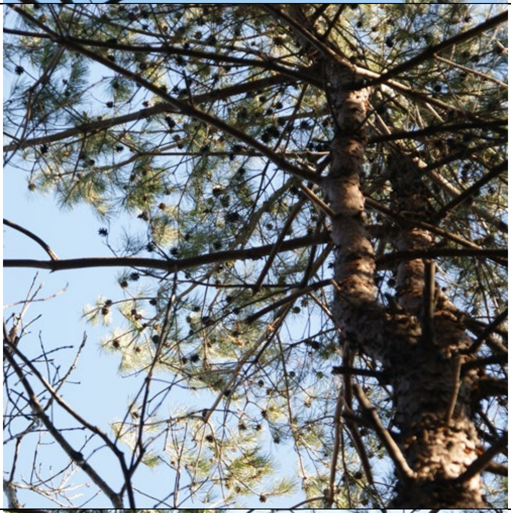
Tree #65	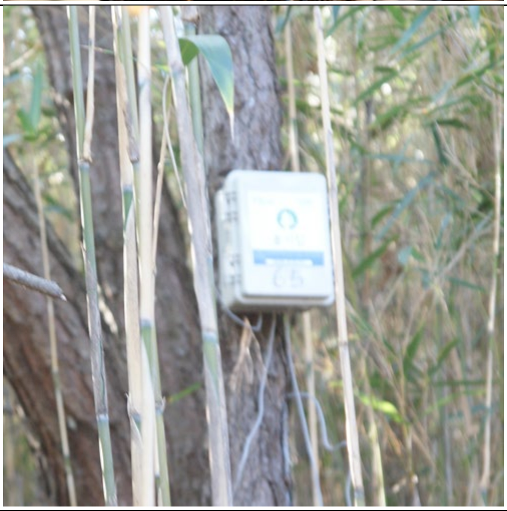	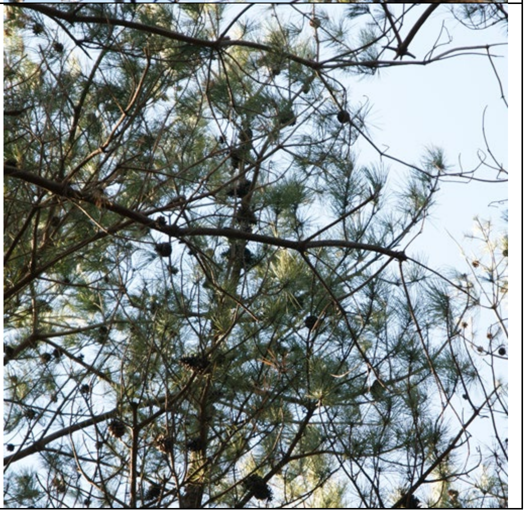

Tree #66	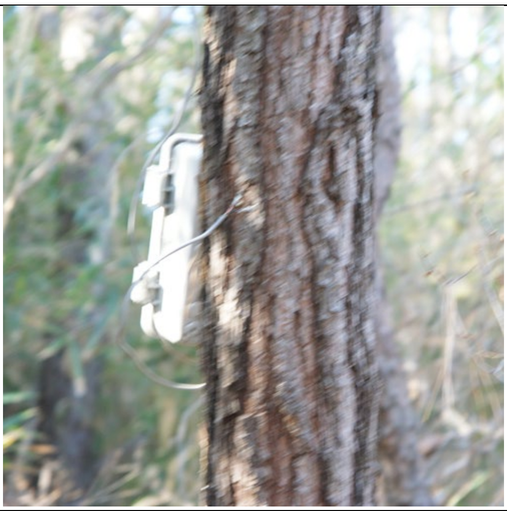	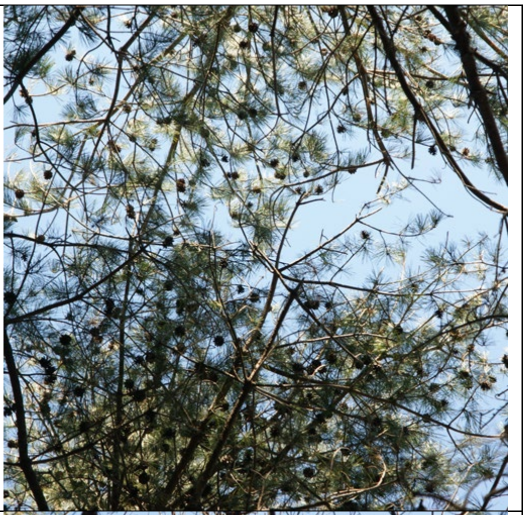
Tree #67	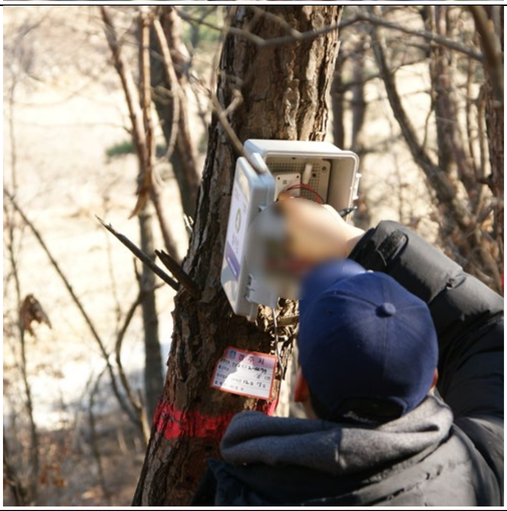	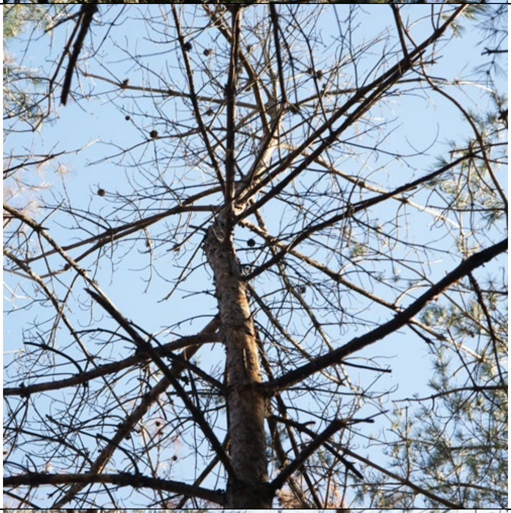
Tree #68	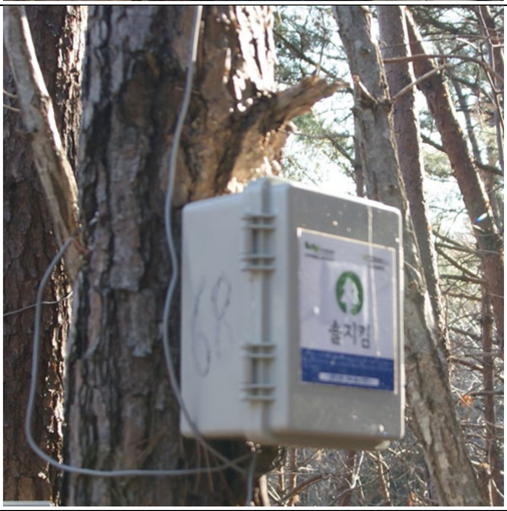	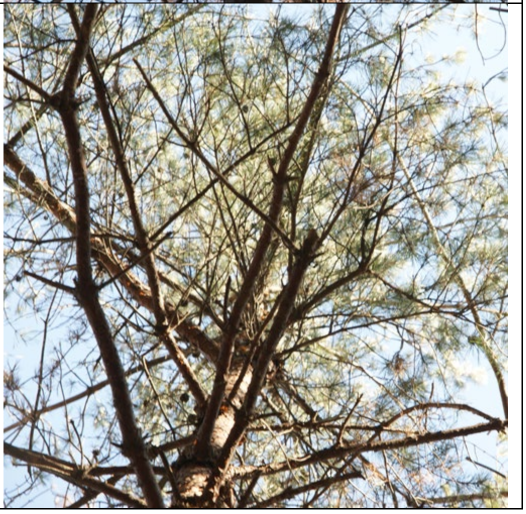

Tree #69	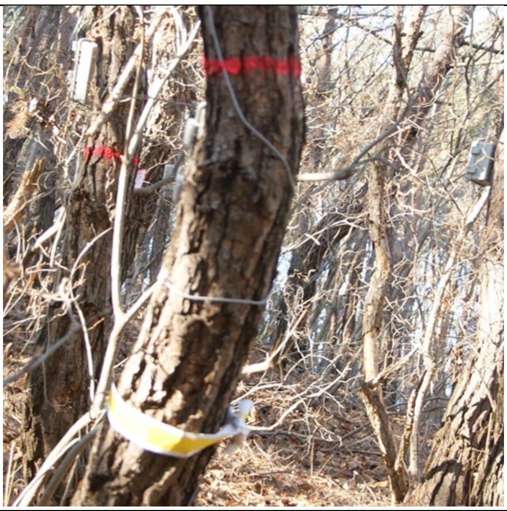	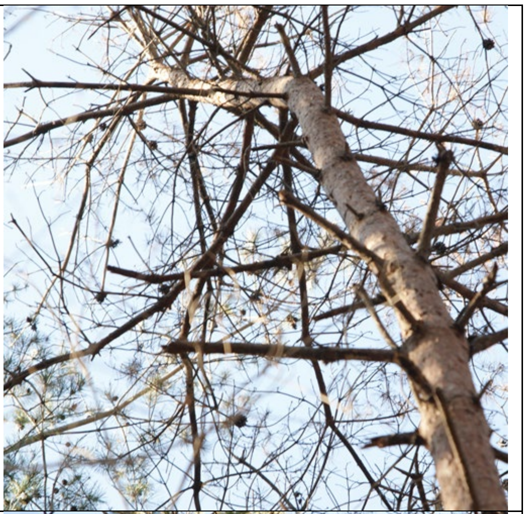
Tree #70	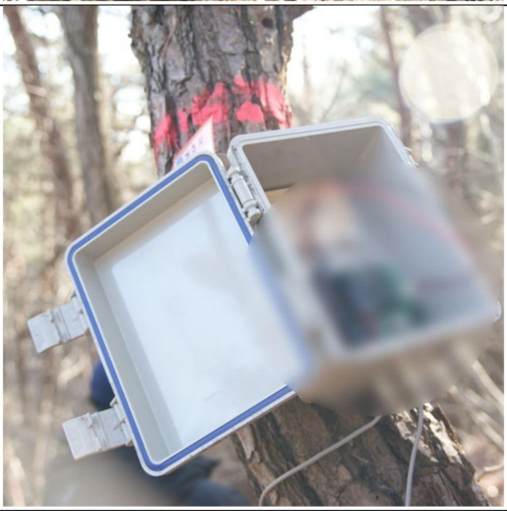	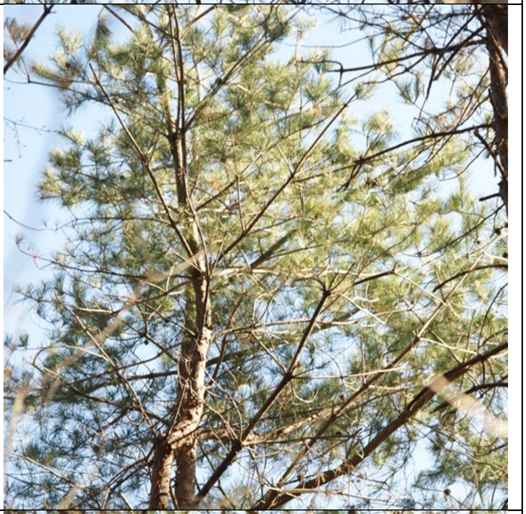
Tree #71	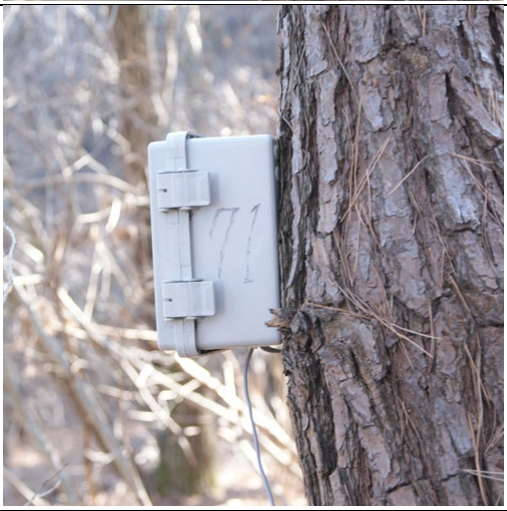	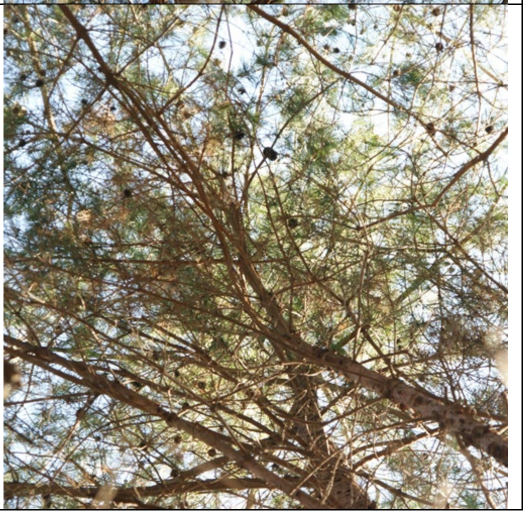

Tree #72	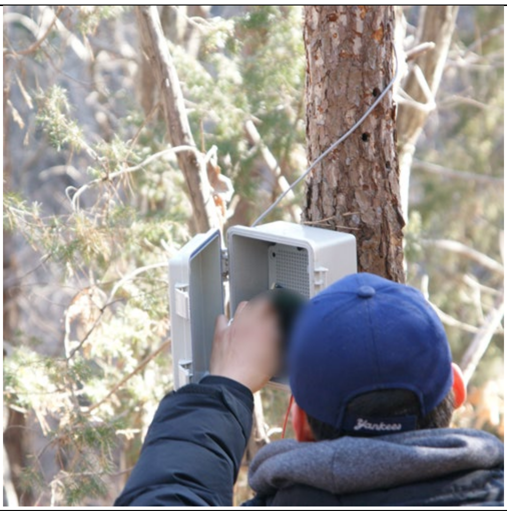	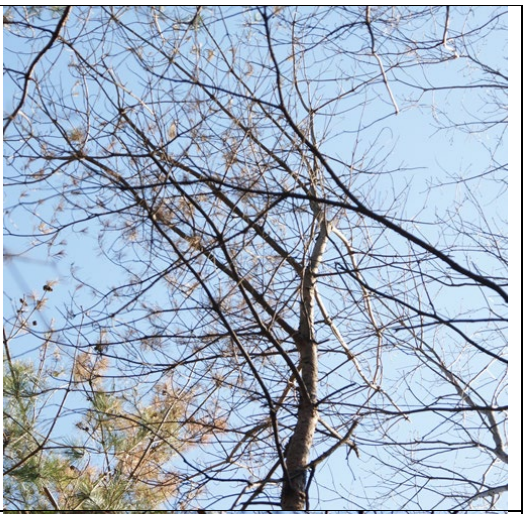
Tree #73	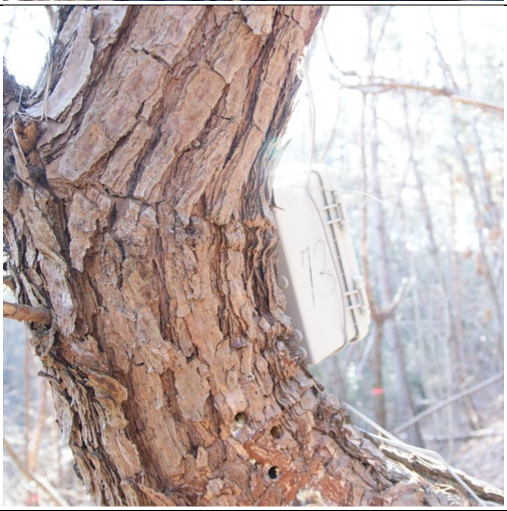	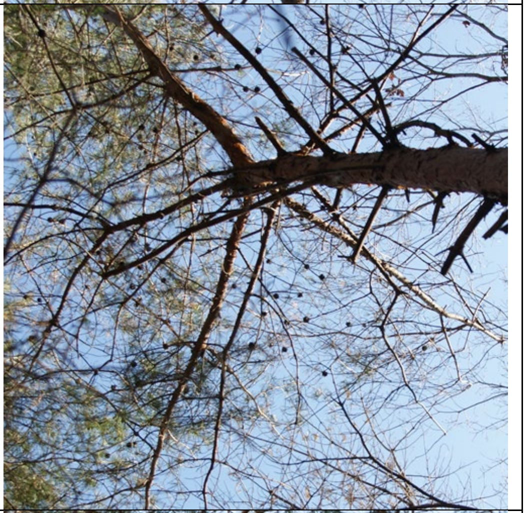
Tree #74	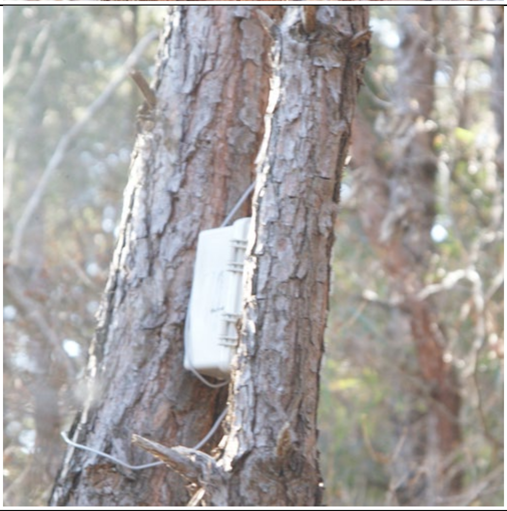	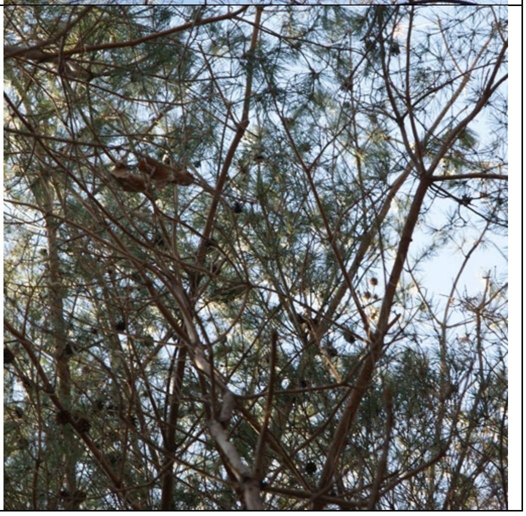

Tree #75	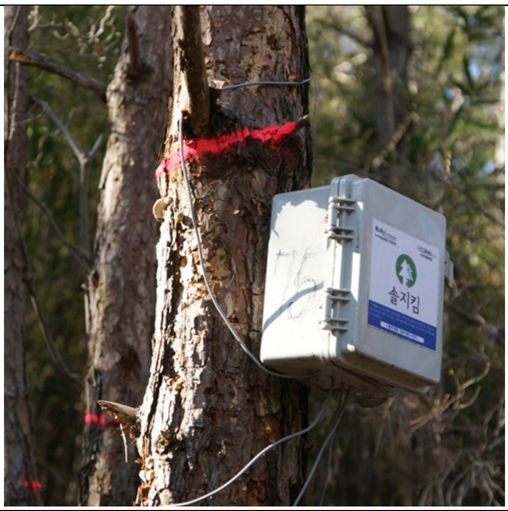	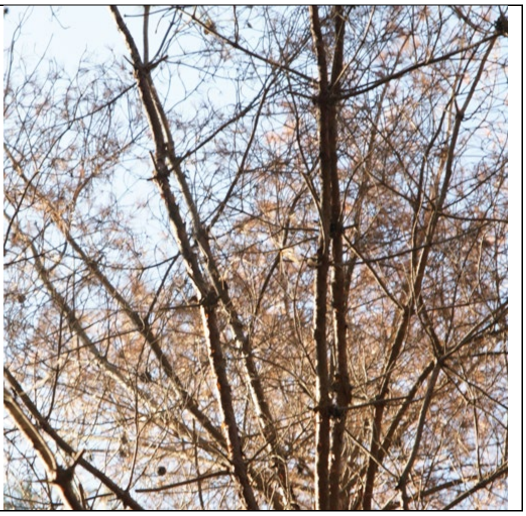
