# Supplementary material for: Diagnosis of pine wilt disease using remote wireless sensing
Source: PLoS One. 2021 Sep 24;16(9):e0257900. doi: 10.1371/journal.pone.0257900 (PMC8462718; doi:10.1371/journal.pone.0257900)

S2 File. MATLAB source code for discriminant analysis

```
%% Draw scatter chart
clear;
load ('x and y.mat')
SL = X(: ,1);
SW = X(:, 2);
group = Y;
h1 = gscatter(SL,SW,group,'rb');

%% Perform Linear
[X,Y] = meshgrid(linspace(0,8),linspace(0,8));
X = X(:); Y = Y(:);
[C,err,P,logp,coeff] = classify([X Y],[SL SW], group,'linear');

%% visualize Linear
hold on;
gscatter(X,Y,C,'rb','.',1,'off');
K = coeff(1,2).const;
L = coeff(1,2).linear;
% Function to compute K + L*v + v'*Q*v for multiple vectors
% v=[x;y]. Accepts x and y as scalars or column vectors.
f = @(x,y) K + L(1)*x + L(2)*y;
h2 = fimplicit(f,[0 8 0 8]);
axis([0 8 0 8]);
intercept = -K/L(2)
slope = -L(1)/L(2)
```

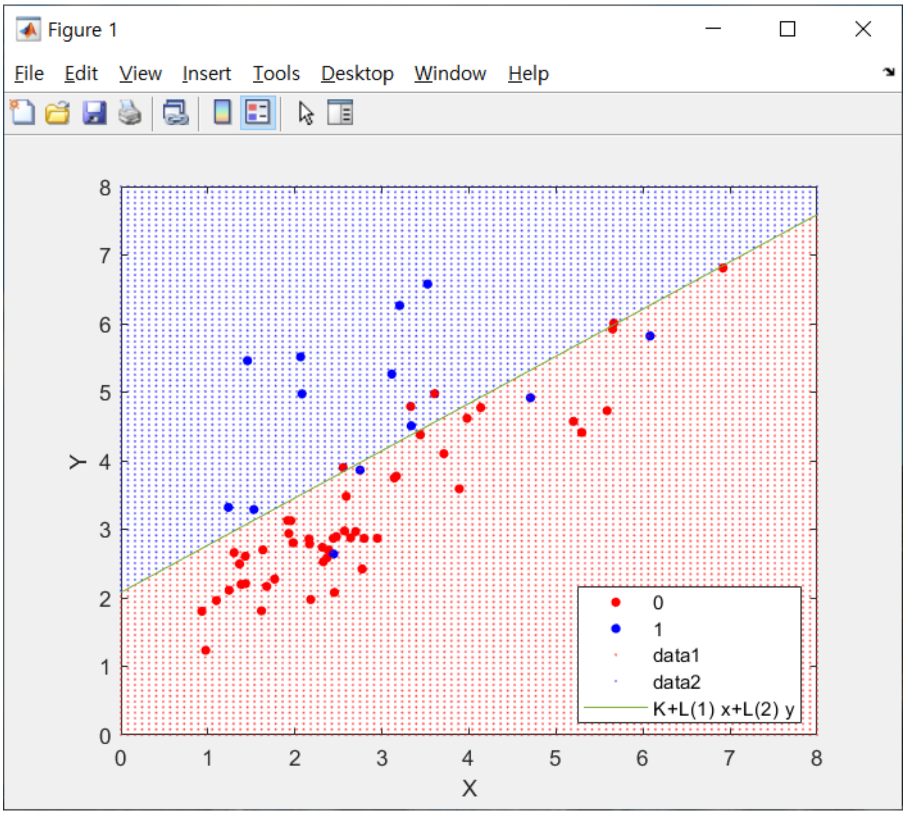

Supplement: S2 File — (PDF) [file pone.0257900.s006.pdf]
